# Identification of the Calmodulin-Binding Domains of Fas Death Receptor

**DOI:** 10.1371/journal.pone.0146493

**Published:** 2016-01-06

**Authors:** Bliss J. Chang, Alexandra B. Samal, Jiri Vlach, Timothy F. Fernandez, Dewey Brooke, Peter E. Prevelige, Jamil S. Saad

**Affiliations:** Department of Microbiology, University of Alabama at Birmingham, Birmingham, AL, 35294, United States of America; The Francis Crick Institute, UNITED KINGDOM

## Abstract

The extrinsic apoptotic pathway is initiated by binding of a Fas ligand to the ectodomain of the surface death receptor Fas protein. Subsequently, the intracellular death domain of Fas (FasDD) and that of the Fas-associated protein (FADD) interact to form the core of the death-inducing signaling complex (DISC), a crucial step for activation of caspases that induce cell death. Previous studies have shown that calmodulin (CaM) is recruited into the DISC in cholangiocarcinoma cells and specifically interacts with FasDD to regulate the apoptotic/survival signaling pathway. Inhibition of CaM activity in DISC stimulates apoptosis significantly. We have recently shown that CaM forms a ternary complex with FasDD (2:1 CaM:FasDD). However, the molecular mechanism by which CaM binds to two distinct FasDD motifs is not fully understood. Here, we employed mass spectrometry, nuclear magnetic resonance (NMR), biophysical, and biochemical methods to identify the binding regions of FasDD and provide a molecular basis for the role of CaM in Fas–mediated apoptosis. Proteolytic digestion and mass spectrometry data revealed that peptides spanning residues 209–239 (Fas-Pep1) and 251–288 (Fas-Pep2) constitute the two CaM-binding regions of FasDD. To determine the molecular mechanism of interaction, we have characterized the binding of recombinant/synthetic Fas-Pep1 and Fas-Pep2 peptides with CaM. Our data show that both peptides engage the N- and C-terminal lobes of CaM simultaneously. Binding of Fas-Pep1 to CaM is entropically driven while that of Fas-Pep2 to CaM is enthalpically driven, indicating that a combination of electrostatic and hydrophobic forces contribute to the stabilization of the FasDD–CaM complex. Our data suggest that because Fas-Pep1 and Fas-Pep2 are involved in extensive intermolecular contacts with the death domain of FADD, binding of CaM to these regions may hinder its ability to bind to FADD, thus greatly inhibiting the initiation of apoptotic signaling pathway.

## Introduction

Apoptosis, also known as programmed cell death, is a strictly regulated process and is a vital component of many processes including normal cell turnover and proper functioning of the immune system. Alteration in apoptosis balance (enhancement or diminishment) is linked to various human diseases such as autoimmune and neurodegenerative disorders, and several types of cancers.[1] The apoptotic pathway is normally initiated by cell surface death receptors such as Fas (also called CD95/Apo1), belonging to the tumor necrosis factor (TNF) receptor family.[2–4] Apoptosis is initiated when the ectodomain of Fas binds to its conjugate ligand, FasL. Fas–FasL binding induces local structural changes in Fas, which allows for a subsequent interaction between the intracellular death domain (DD) of Fas (FasDD) and an analogous DD belonging to Fas-associated death domain (FADD).[2, 5–7] These interactions trigger a cascade of subsequent interactions that lead to activation of caspases, which can be achieved through two distinct but ultimately converging apoptotic pathways, extrinsic and intrinsic.[8] Binding of both FasDD and procaspase-8 to FADD form the core of death-inducing signaling complex (DISC). Activated caspase-8 then cleaves and activates caspase-3, -6 and -7, which target cellular substrates and ultimately execute cell death.[8, 9]

The DISC formation is a critical step in regulating the Fas–mediated apoptotic pathway. Besides FasDD, FADD and procaspase-8, the DISC assembly also includes procaspase-10 and the caspase-8/10 regulator c-FLIP (FADD-like interleukin-1β–converting enzyme (FLICE)-inhibitory protein). Previous studies have shown that calmodulin (CaM) is also recruited to the DISC in cholangiocarcinoma [10–16] and pancreatic cancer cells.[17] The level of CaM recruited into the DISC is increased upon Fas stimulation.[12] Inhibition of CaM activity in the DISC stimulates apoptosis significantly.[10, 14, 18] Based on genetic, biochemical and in vivo data it was suggested that CaM acts as regulator of the apoptotic pathway by interacting with FasDD, thus inhibiting its interaction with FADD.[10–16] CaM is known as a major regulator of Ca^2+^-dependent signaling in all eukaryotic cells [19–24] and plays a vital role in the control of many physiological processes such as cell proliferation, apoptosis, protein folding, autophagy, gene expression, metabolic homeostasis and many others.[25] The structure, function and mechanism of CaM binding to target proteins have been extensively studied over the last two decades.[19, 20, 23, 26] CaM possesses an exceptional versatility in structural rearrangement upon binding to targets.[21, 23, 27, 28] Understanding its binding is often complicated by the diversity of target proteins sequences. The CaM protein undergoes major structural rearrangements upon binding to Ca^2+^, resulting in the opening of large hydrophobic binding pockets on the surface of N- and C-lobes (CaM-N and CaM-C, respectively).[20, 23, 24] Ca^2+^/CaM adopts a “dumbbell-like” architecture with the N- and C-terminal lobes connected by a flexible central linker.[29–31] It was found that CaM regulatory role in the apoptotic pathway and its interaction with FasDD are dependent on calcium.[12]

Previous mutagenesis studies suggested that the Ca^2+^/CaM binding site in FasDD is located between residues 214–254 (numbered 230–270 in that study).[11] We have recently provided compelling evidence for the formation of a ternary Ca^2+^/CaM–FasDD complex with two Ca^2+^/CaM molecules binding simultaneously to FasDD.[32] We have also shown that both lobes of Ca^2+^/CaM are important for FasDD binding. These results provided new insight into the mechanism of binding; however, how CaM can bind to two distinct motifs on FasDD is not understood. Our attempt to identify the interaction regions by NMR was hampered by the twin problems, the large size of the complex and the intermediate chemical exchange rate on the NMR scale, which led to severe broadening of the NMR signals precluding unambiguous identification of residues critical for binding.[32]

**Fig 1:**
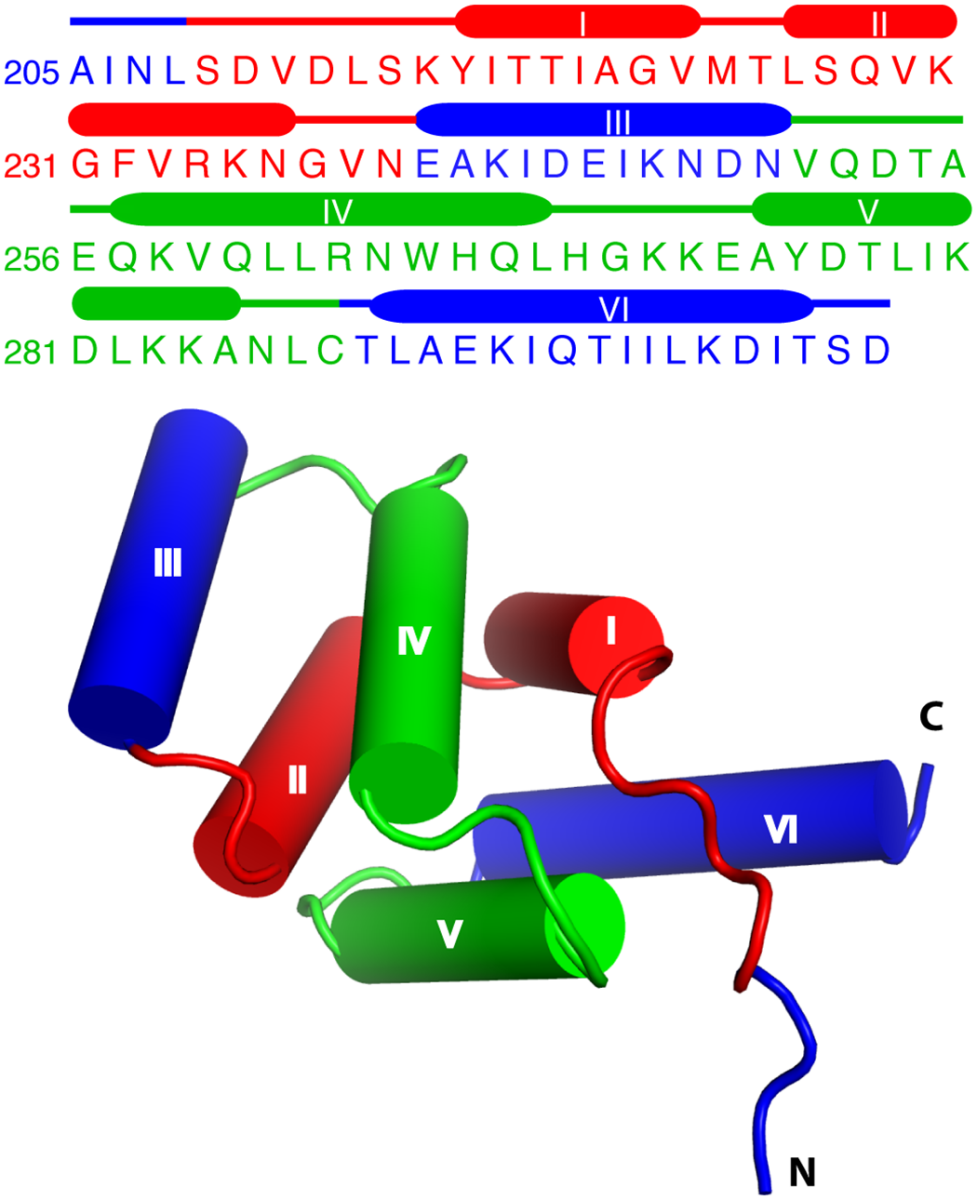
FasDD sequence and structure. Protein sequence, secondary structure and a cartoon representation of the three-dimensional structure of FasDD (PDB ID: 1DDF). The CaM-binding regions (Fas-Pep1 and Fas-Pep2) are highlighted in red and green, respectively.

Here, we employed mass spectrometry, nuclear magnetic resonance (NMR), biophysical, and biochemical assays to identify the binding regions of FasDD and to characterize the interaction interface with Ca^2+^/CaM. Proteolytic digestion aided by mass spectrometry revealed that the two Ca^2+^/CaM-binding domains of FasDD are located between amino acids 209–239 and 251–288 (which will be referred to as Fas-Pep1 and Fas-Pep2 throughout this work, Fig 1). Elucidation of the structural determinants of FasDD–CaM interaction and the mechanism of inhibition will be critical to understanding the precise molecular mechanism of Fas–mediated apoptosis, which may help in the development of new anticancer therapeutic strategies.

## Materials and Methods

### Plasmid construction

A molecular clone harboring the full-length FasDD gene was a kind gift from Dr. Jay McDonald (The University of Alabama at Birmingham). We used the numbering of FasDD amino acids as described (Fig 1).[3, 33] A gene encoding for Fas-Pep2 (residues 251–288) was PCR-amplified from the full-length FasDD gene and ligated to the 3’-end of small ubiquitin-like modifier (SUMO) gene via BamHI and XhoI sites within a pET28 vector. A DNA sequence encoding for a His_6_ tag is appended on the 5’-end of the SUMO gene.

### Protein expression and purification

FasDD, Ca^2+^/CaM, Ca^2+^/CaM-N and Ca^2+^/CaM-C proteins were prepared as described.[32, 34] Ca^2+^/CaM-N protein concentration was measured using bicinchoninic (BCA) protein assay (Thermo Scientific) because it has zero extinction coefficient at 280 nm. The His_6_-SUMO-Fas-Pep2 protein was produced in *E*. *coli* (BL21-CodonPlus(DE3)-RIL strain). To make an unlabeled sample of Fas-Pep2, 20 mL of starter cells harboring SUMO-Fas-Pep2 plasmid were grown overnight at 37°C in lysogeny broth (LB) media containing kanamycin (50 mg/L). Next day, cells were transferred to 1 L of LB media and grown until O.D._600_ of ~0.6–0.7 before induction with 1 mM IPTG. Cells were induced for 4 hours, harvested, spun down at 5,000 rpm and stored overnight at -80°C. Next day, the cell pellet was resuspended in 40 mL of lysis buffer containing 50 mM Tris•HCl (pH 8), 300 mM NaCl and 5 mM 2-mercaptoethanol. Cells were sonicated and cell lysate was spun down at 17,000 rpm for 30 min. The supernatant containing His_6_-SUMO-Fas-Pep2 protein was loaded on nickel resin (Thermo Scientific) and protein was washed and eluted by chromatography techniques using a buffer containing 300 mM imidazole. Fractions were dialyzed overnight in a buffer containing 25 mM Tris•HCl (pH 8) and 150 mM NaCl. The His_6_-SUMO-Fas-Pep2 protein was cleaved by SUMO protease and Fas-Pep2 peptide further purified via nickel affinity and gel filtration chromatography methods. To make uniformly ^15^N- and ^15^N,^13^C-labeled Fas-Pep2 samples, cells were grown in minimal media containing ^15^NH_4_Cl and/or ^13^C-glucose as the sole nitrogen and carbon sources to produce ^15^N- and/or ^13^C-labeled proteins, respectively. The molecular mass of Fas-Pep2 was confirmed by mass spectrometry. Synthetic Fas-Pep1 and FasDD peptides spanning residues 214–238 (FasDD(214–238)) and 224–238 (FasDD(224–238)) were purchased with > 95% purity and used as received (Genscript, Piscataway, NJ). Because the solubility in aqueous buffers was low (~50 μM), 4 mM stock solution of Fas-Pep1 was prepared in 100% DMSO-d_6_. A stock solution of FasDD(214–238) at 8 mM was prepared in 60% (50 mM Tris-d11 (pH 7.0), 100 mM NaCl and 5 mM CaCl_2_) and 40% DMSO-d_6_. A stock solution of FasDD(224–238) at 6.5 mM was prepared in 50 mM Tris-d11 (pH 7.0), 100 mM NaCl and 5 mM CaCl_2_. All experiments were conducted in the presence of calcium.

### Proteolysis assay

Proteolytic digestion reactions were conducted on highly pure samples of FasDD, Ca^2+^/CaM and FasDD–Ca^2+^/CaM complex in a buffer containing 50 mM HEPES (pH 7.0), 50 mM NaCl and 5 mM CaCl_2_. The complex was made at 2:1 (Ca^2+^/CaM:FasDD) ratio. Protein samples were then subjected to limited proteolysis by addition of subtilisin (from *Bacillus licheniformis*, Sigma-Aldrich) at 1:500 (enzyme:complex) molar ratio. All digestion experiments were performed at 4°C and were monitored for 24 hours via SDS-PAGE and Coomassie blue staining.

### Mass spectrometry

In order to identify the FasDD peptides in the digested samples, Ca^2+^/CaM–FasDD digests were separated with reverse phase HPLC (1100 Agilent) coupled to an electrospray ionization quadrupole TOF mass spectrometer (Waters Q-TOF Premier). Digests were loaded and quickly washed on a 100 x 2mm C18 Monolithic column (Phenomenex, Torrance, CA) with 5% acetonitrile (ACN) + 0.1% formic acid (FA) (v/v) for 0.5 min at a flow rate of 0.5 mL/min. After washing the loaded sample, peptides were eluted using a 10 min ACN step gradient (0–5 min 5–30% ACN +0.1% FA, 5–7 min 30–95% ACN + 0.1% FA) and electrosprayed into an ESI-TOF mass spectrometer (Waters QTOF Premier). The sample contained mostly large peptides, which eluted from 5.5 to 7.0 min. The Ca^2+^/CaM protein eluted as an intact polypeptide. Eluted peptides were then selected for MS/MS analysis using CID fragmentation. Survey and MS/MS data were analyzed by Waters ProteinLynx Global Server (2.5.2) using a custom database containing both Ca^2+^/CaM and FasDD. Peptides were then identified by MS/MS and accurate mass measurements (S1 Table).

### Gel filtration assay

The mobility of Ca^2+^/CaM complexes with Fas-Pep1 and Fas-Pep2 was analyzed by a gel filtration assay. Briefly, 0.5 mL of protein samples (~50–150 μM) were run on a HiLoad Superdex 75 (10/300 GL) column (GE Healthcare) in a buffer containing 50 mM Tris (pH 7.0), 100 mM NaCl and 5 mM CaCl_2_. Protein fractions were analyzed by SDS-PAGE. A low molecular weight calibration kit (GE Healthcare) was used to determine the approximate molecular weight of complexes.

### Isothermal titration calorimetry (ITC)

Thermodynamic parameters of Ca^2+^/CaM binding to Fas-Pep1 and Fas-Pep2 peptides were determined using an Auto-iTC_200_ microcalorimeter (Malvern Instruments). ITC experiments were performed on protein samples in 50 mM HEPES (pH 7.0), 100 mM NaCl, and 5 mM CaCl_2_. Ca^2+^/CaM at 450 or 195 μM was titrated into the cell sample containing 25 or 17 μM of Fas-Pep1 or Fas-Pep2, respectively. Heat of reaction of Ca^2+^/CaM was measured over 19 injections at 25°C for Fas-Pep1 or 35°C for Fas-Pep2. Heat of dilution was measured by titrating Ca^2+^/CaM into buffer. Data analysis was performed using the Microcal Origin package (ver. 8.1). Baseline corrections were performed by subtracting heat of dilution from the raw Ca^2+^/CaM-peptide titration data. Binding curves were analyzed and dissociation constants (*K*_d_) were determined by nonlinear least-square fitting of the baseline-corrected data. The formula used to fit the data as one binding site is:

$$\Delta Q\left(\text{i}\right)=Q\left(\text{i}\right)+(dV\text{i}/V_{o})[(Q\left(\text{i}\right)+Q\left(\text{i}-1\right))/2]–Q(\text{i}–1)$$

where Δ*Q*(i) is the heat released at i^th^ injection, *Q*(i) is the total heat content of the solution, *dV*_i_ is injection volume, and *V*_*o*_ is total volume. Three replicate titration experiments were typically performed for each peptide.

### Circular dichroism (CD) spectroscopy

CD spectra were acquired on a Jasco J815 spectropolarimeter at 25°C from 260 to 185 nm. Scanning rate was set to 50 nm/min. Loading concentrations were ~16–20 μM for free peptides, 18 μM for free Ca^2+^/CaM and 10 μM for the complexes in a buffer containing 10 mM HEPES (pH 7), 50 mM KCl, and 2.5 mM CaCl_2_. The background signal from the buffer solution was subtracted from each spectrum. Ca^2+^/CaM complexes with peptides were run on gel filtration column (as described above) to ensure high purity and homogeneity prior to collection of the CD spectra.

### NMR spectroscopy

NMR data were collected at 35°C on a Bruker Avance II (700 MHz ^1^H) spectrometer equipped with a cryogenic triple-resonance probe, processed with NMRPIPE [35] and analyzed with NMRVIEW [36] or CCPN Analysis [37]. All NMR samples were prepared in a buffer containing 50 mM Tris-d11 (pH 7.0), 100 mM NaCl and 5 mM CaCl_2_. ^15^N-labeled Ca^2+^/CaM samples used for NMR titration data were at 100–150 μM. Peptide samples used for titration into ^15^N-labeled Ca^2+^/CaM, Ca^2+^/CaM-N and Ca^2+^/CaM-C samples were at 2–4 mM. For signal assignments of the complexes, ^13^C-, ^15^N-, or ^13^C-/^15^N-labeled Ca^2+^/CaM at ~400–500 μM was mixed with unlabeled Fas-Pep1 (in 100% DMSO-d_6_) or Fas-Pep2 at 1.5:1 peptide:Ca^2+^/CaM. For Ca^2+^/CaM–Fas-Pep1 complex, the sample was washed with NMR buffer containing 50 mM Tris-d11 (pH 7.0), 100 mM NaCl and 5 mM CaCl_2_ to remove residual DMSO-d_6_. The backbone atom resonances of Ca^2+^/CaM complexes with Fas-Pep1 and Fas-Pep2 were assigned using HNCA, HN(CO)CA, HNCACB, HNCO, HN(CO)CACB and ^15^N-edited NOESY-HSQC and TOCSY-HSQC experiments. These experiments have been also collected on ^15^N-, or ^13^C-/^15^N-labeled Fas-Pep2 in complex with unlabeled Ca^2+^/CaM to confirm the α-helical character of Fas-Pep2 when bound to Ca^2+^/CaM. The chemical shifts of Ca^2+^/CaM-bound Fas-Pep2 were used to predict its order parameters and secondary structure content in TALOS+.[38] Combined ^1^H-^15^N chemical shift changes were calculated as Δδ_HN_ = [(Δδ_H_)^2^+(Δδ_N_/5)^2^]^1/2^, where Δδ_H_ and Δδ_N_ are the ^1^H and ^15^N chemical shift changes, respectively. *K*_d_ values were calculated by non-linear least-square fitting algorithm in Origin software (OriginLab, Northampton, MA) using the equation:

$$\Delta \delta_{\text{HN}}=\Delta \delta_{\text{HN}}{}^{\text{max}}((K_{\text{d}}+{\text{L}}_{0}+{\text{P}}_{0})-{\left({\left(K_{\text{d}}+{\text{L}}_{0}+{\text{P}}_{0}\right)}^{2}-4*{\text{P}}_{0}*{\text{L}}_{0}\right)}^{0.5})/\left(2*{\text{P}}_{0}\right)$$

where Δδ_HN_^max^ is the chemical shift change between complex and free protein, L_0_ total concentration of ligand, and P_0_ total concentration of protein.

### NMR data deposition

The chemical shifts of Ca^2+^/CaM in complex with Fas-Pep1 and Fas-Pep2 have been deposited in the Biological Magnetic Resonance Bank with the accession codes 26626 and 26627, respectively.

## Results

To identify the Ca^2+^/CaM-binding regions in large proteins is not a simple task. To predict the Ca^2+^/CaM-binding site, a web-based tool is often used to provide favorable scores based on multiple criteria including hydropathy, α-helical propensity, hydrophobic residue content, residue charge, residue weight, helical class and occurrence of particular residues.[39] However, this method can still provide biased results. Analysis of the FasDD protein sequence using this method yielded favorable scores for a region spanning residues 222–240, which forms an α-helix (α2) and a short loop (Fig 1). Residues 282–299 are also predicted to form a second, but less favorable, Ca^2+^/CaM-binding. A recent study [40] has shown that FasDD(214–238) binds to Ca^2+^/CaM with a much weaker affinity (*K*_d_ = 19.5 μM) than that observed for the full-length FasDD protein (*K*_d_ ~2 μM). The x-ray structure of Ca^2+^/CaM in complex with FasDD(214–238) revealed that only residues 214–227 are ordered and bound to Ca^2+^/CaM; a defined electron density for residues 228–238 was not observed. In this x-ray structure, Ca^2+^/CaM adopts a compact ellipsoidal structure whereby both domains of CaM are wrapped around the FasDD peptide, which adopts an α-helical conformation.[40] This result is in contrast with the result obtained by the web-based tool. The relatively weak binding affinity of this FasDD peptide suggests that it may not be a true representative of the CaM-binding region of FasDD. Because of these controversial results and because none of the previous experimental studies suggested the presence of a second CaM-binding site in FasDD,[11, 41] we have employed mass spectrometry, NMR, biochemical, and biophysical approaches to precisely identify the binding domains of FasDD.

### Limited proteolysis reveals the CaM-binding domains of FasDD

**Fig 2:**
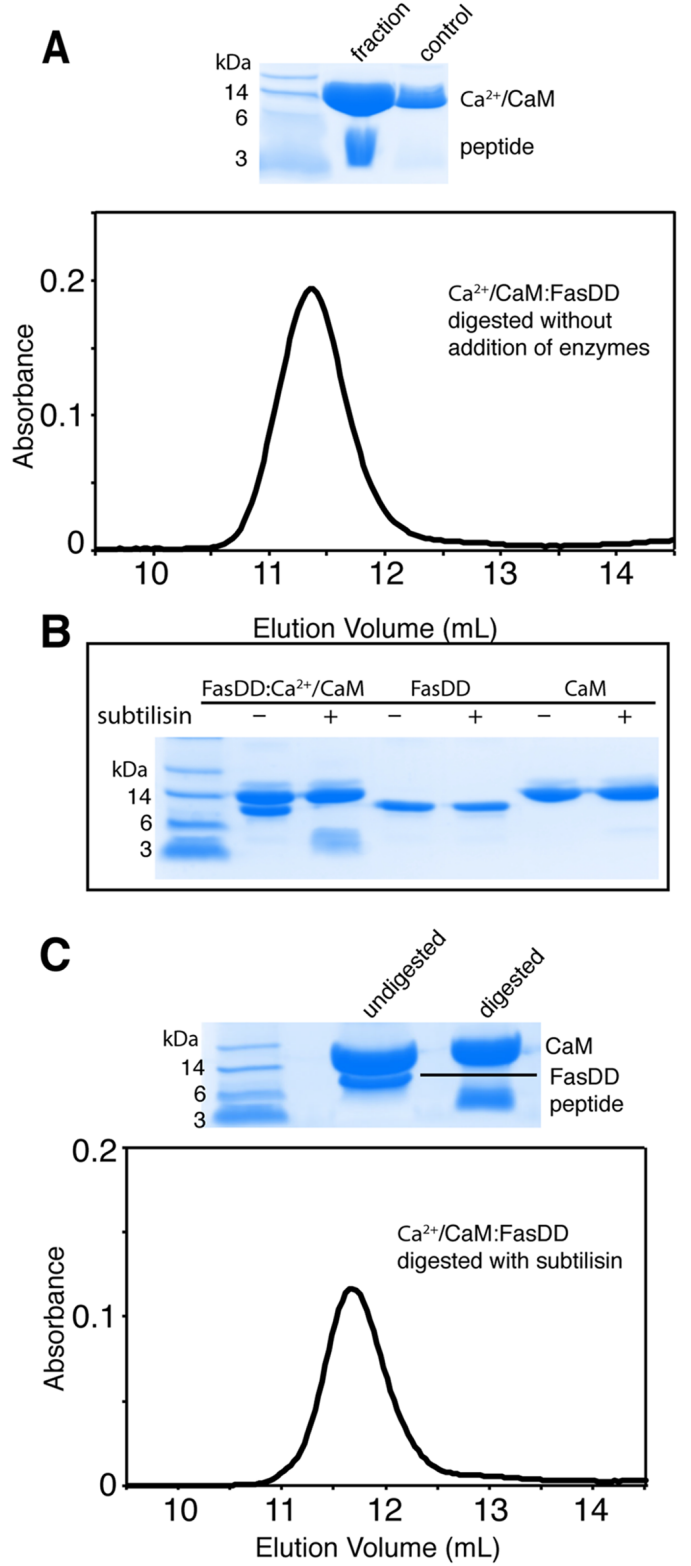
Proteolysis assay and gel filtration of Ca^2+^/CaM–FasDD digests. (**A**) Gel filtration and SDS-PAGE of the proteolysis product of a 2:1 Ca^2+^/CaM:FasDD sample left at 4°C for one week. The SDS-PAGE data show that the degraded FasDD protein gave rise to ~5 kDa fragment(s). The Ca^2+^/CaM protein, however, remained stable. Analysis of the digestion products by mass spectrometry confirmed the identity of the FasDD peptides that are resistant to proteolysis. The most abundant peptides are located in the N-terminus (205–238, 205–239, and 205–240) and in the C-terminus (251–288, 259–288 and 262–288). (**B**) SDS-PAGE of FasDD, Ca^2+^/CaM and Ca^2+^/CaM:FasDD complex without or with added subtilisin at 1:500 (enzyme:protein) 24 hours after initiation of the reaction. Unbound FasDD and Ca^2+^/CaM were stable in the presence of subtilisin. (**C**) Gel filtration and SDS-PAGE data of the proteolysis product of a 2:1 Ca^2+^/CaM:FasDD sample treated with subtilisin for 24 hours. Similar to our observation in A, the SDS-PAGE data show a band at ~5 kDa eluting with Ca^2+^/CaM. FasDD fragments were identified by mass spectrometry (S1 Table).

We sought to determine the CaM-binding regions of FasDD by utilizing a proteolytic digestion assay followed by analysis with mass spectrometry. Our initial attempt involved an un-induced proteolysis reaction with no proteases added to the sample. A Ca^2+^/CaM–FasDD complex was made in a 2:1 (CaM:FasDD) ratio and left at 4°C for one week. The stability of the complex was monitored by SDS-PAGE. We observed that with time, the FasDD protein degraded and gave rise to stable and proteolysis resistant ~5 kDa FasDD fragment(s). The Ca^2+^/CaM protein, however, remained intact and stable. The sample containing Ca^2+^/CaM and degraded fragments was run through the gel filtration column to determine whether any of the FasDD fragments eluted with Ca^2+^/CaM. Small Ca^2+^/CaM-bound FasDD fragments were detected by SDS-PAGE (Fig 2A). Analysis of the digestion products by mass spectrometry revealed that several FasDD peptides were resistant to proteolysis. As identified by both exact mass measurements and tandem mass spectrometric sequencing, the most abundant peptides are located in the N-terminus (205–238, 205–239, and 205–240) and in the C-terminus (251–288, 259–288 and 262–288). Sequence analysis of the FasDD fragments indicated cleavage by subtilisin, which was unavoidably present as a persistent minor contamination during protein preparation. Interestingly, the unbound CaM and FasDD control samples were stable and not prone to degradation when left at 4°C for one week, indicating that Ca^2+^/CaM binding to FasDD may have induced conformational changes in the FasDD protein exposing regions susceptible to protease digestion.

In the second approach, we conducted proteolytic digestion on the Ca^2+^/CaM–FasDD complex using subtilisin (Fig 2B). A sample made with a 2:1 (Ca^2+^/CaM:FasDD) ratio was subjected to proteolysis by addition of subtilisin at 1:500 (enzyme:complex) molar ratio. Similar to the digestion without added protease, FasDD was cleaved to fragment(s) of ~5 kDa while Ca^2+^/CaM remained intact. Unbound FasDD and Ca^2+^/CaM were resistant to proteolysis by subtilisin (Fig 2B). When the digestion products were run through a gel filtration column, FasDD fragments of ~5 kDa eluted with the Ca^2+^/CaM protein (Fig 2C). Mass spectrometry data revealed that the most abundant fragments are located between residues 205 and 241 (S1 Table). A fragment corresponding to amino acids 253–273 was also detected. This fragment is shorter than that detected when the Ca^2+^/CaM–FasDD complex was left at 4°C for one week, possibly because of a rapid digestion by subtilisin at the experimental conditions. Taken together, our results demonstrate that the shortest Ca^2+^/CaM binding sites of FasDD are located between residues 209–236 and 251–288. Because residue V238 of FasDD has been previously implicated in CaM binding,[11] and because the mass spectrometry data also show that many of the abundant N-terminal fragments contain this residue, we designed our peptides as residues 209–239 (Fas-Pep1) and 251–288 (Fas-Pep2).

### Mobility characteristics of CaM complexes with FasDD peptides

**Fig 3:**
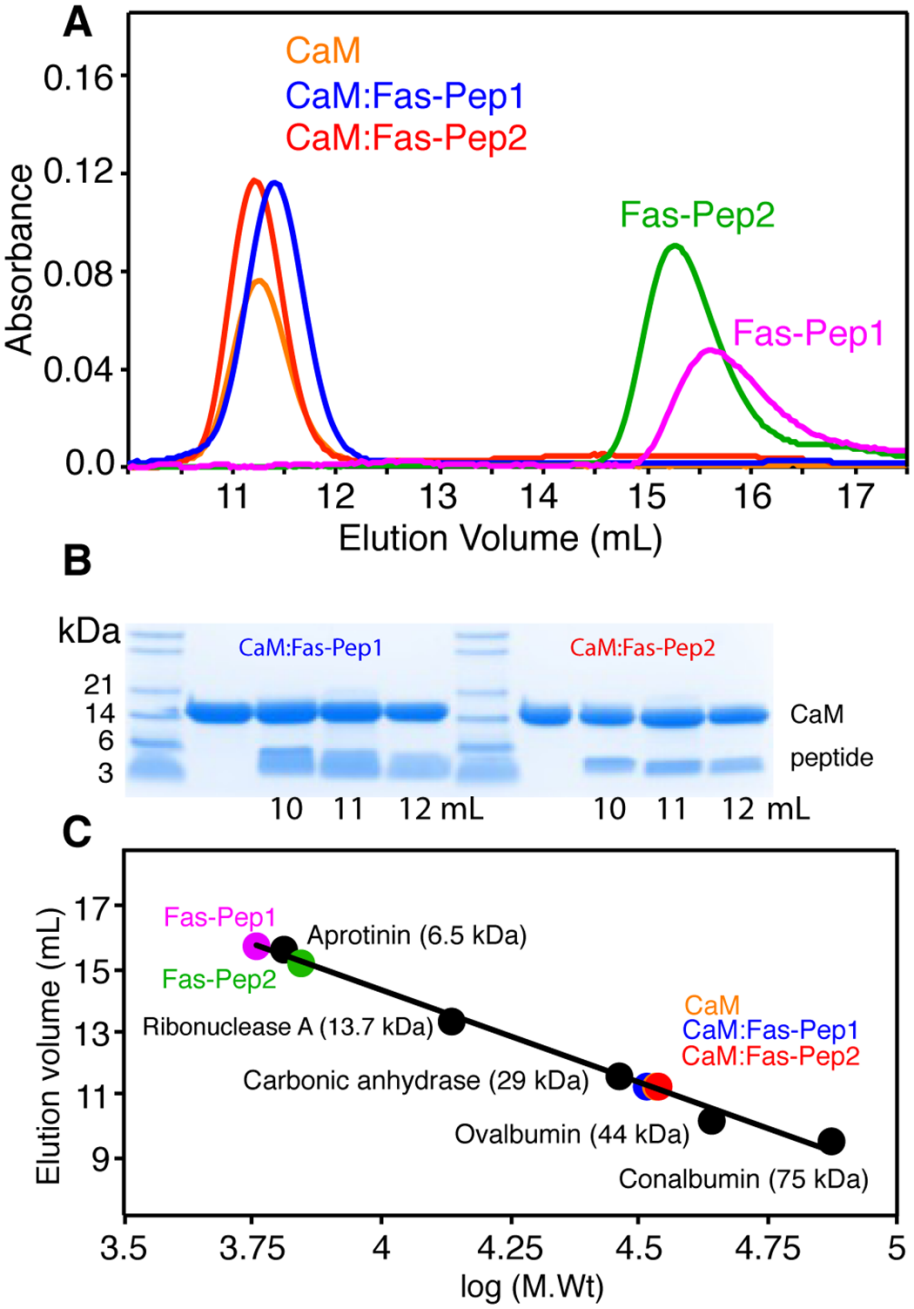
Gel filtration data of Ca^2+^/CaM complexes with recombinant FasDD peptides. (**A**) Gel filtration chromatograms of Ca^2+^/CaM complexes with FasDD peptides using a HiLoad Superdex 75 (10/300 GL) column. (**B**) Complex formation has been confirmed by SDS-PAGE. Two bands of CaM and peptides are clearly observed. Elution of the peptides with CaM indicates direct binding. (**C**) Gel filtration calibration curve with indicated mobility of Ca^2+^/CaM, FasDD peptides and their complexes. The approximate molecular weights of complexes suggest a 1:1 stoichiometry.

Fas-Pep2 has been expressed and purified as a fusion protein with SUMO (see Materials and Methods). Because of some technical difficulties during purification of a recombinant peptide, a synthetic Fas-Pep1 was used in this study. The solution properties of Fas-Pep1 and Fas-Pep2 and their complexes with Ca^2+^/CaM were initially analyzed with size exclusion chromatography assay. Samples were run on a size exclusion column (Superdex 75) under identical buffer conditions. Sample concentrations were at ~50–100 μM. As shown in Fig 3A, the elution volumes of Ca^2+^/CaM, Fas-Pep1 and Fas-Pep2 were at 11.2, 15.6 and 15.2 mL, respectively. Ca^2+^/CaM complexes with Fas-Pep1 and Fas-Pep2 prepared at 1:1 stoichiometry eluted at 11.3 and 11.2 mL, respectively (Fig 3A). No changes in the elution volumes of the Ca^2+^/CaM–Fas-Pep1 and Ca^2+^/CaM–Fas-Pep2 complexes were observed at higher peptide ratio, indicating complete formation of the complex at 1:1 molar ratio. The formation of complexes was confirmed by SDS-PAGE (Fig 3B). Comparison of the elution volumes of the complexes with those obtained for proteins with known molecular weights suggests that complexes between Ca^2+^/CaM and FasDD peptides form at 1:1 stoichiometry (Fig 3C). As we and others have shown previously, Ca^2+^/CaM elutes at a smaller than expected volume due to its elongated dumbbell shape.[32, 42, 43]

### Thermodynamic properties of CaM binding to FasDD peptides

**Fig 4:**
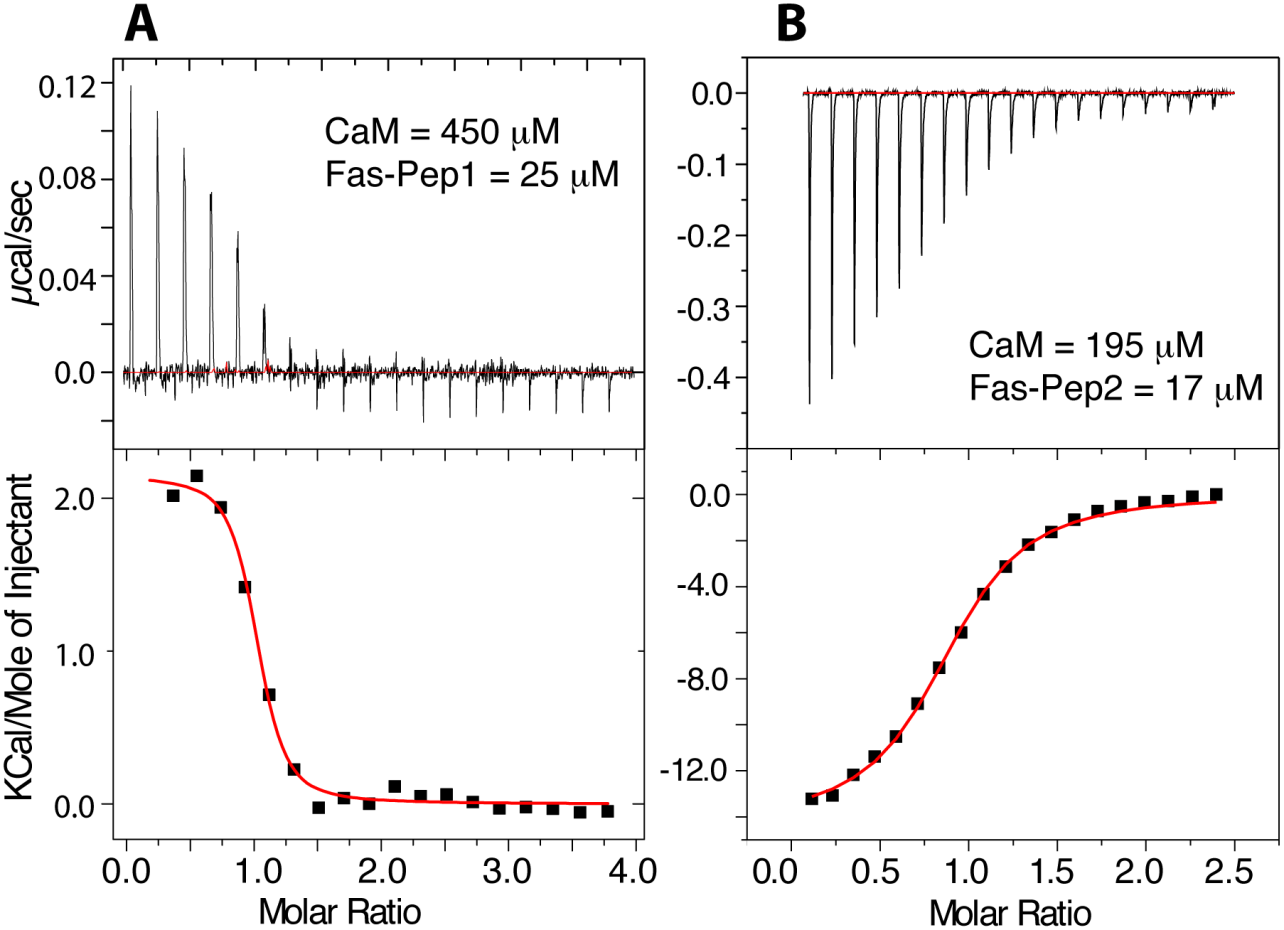
ITC data of Ca^2+^/CaM binding to FasDD peptides. ITC data obtained for titration of (**A**) Ca^2+^/CaM at 450 μM into Fas-Pep1 at 25 μM, and (**B**) Ca^2+^/CaM (195 μM) into Fas-Pep2 at 17 μM. Data fitting afforded *K*_d_ values of 0.3 and 1.1 μM for Fas-Pep1 and Fas-Pep2, respectively.

Although it is recognized that the most frequent mechanism of CaM binding to target proteins involves the hydrophobic surfaces on the N- and C-terminal lobes,[23] electrostatic interactions also contribute to the formation and stabilization of CaM-protein complexes since CaM is acidic and CaM-binding motifs are often basic.[44, 45] The thermodynamic parameters of binding and the relative contribution of hydrophobic vs. electrostatic factors are often assessed by ITC methods, yielding various parameters such as dissociation constant (*K*_d_), stoichiometry (*n*), enthalpy change (Δ*H*°) and entropy change (Δ*S*°). The thermodynamic parameters of Ca^2+^/CaM binding to Fas-Pep1 and Fas-Pep2 were assessed by ITC. As shown in Fig 4, fitting of the ITC data by a single set of identical sites model upon titration of Ca^2+^/CaM at 450 μM into Fas-Pep1 at 25 μM yielded the following thermodynamic parameters: *K*_d_ = 0.3 μM, *n* = 0.95, Δ*H*° = 2.15 kcal/mol and Δ*S*° = 37.1 cal/mol/K. Likewise, fitting of the ITC data by a single set of identical sites model upon titration of Ca^2+^/CaM at 195 μM into Fas-Pep2 at 17 μM yielded the following thermodynamic parameters: *K*_d_ = 1.1 μM, *n* = 0.9, Δ*H*° = -14.2 kcal/mol and Δ*S*° = -18.8 cal/mol/K. Consistent with the gel filtration results, the ITC data clearly show that stoichiometry of binding of each of the peptides to Ca^2+^/CaM is 1:1. Interestingly, as indicated by the enthalpy and entropy factors hydrophobic interactions are important for the formation of Ca^2+^/CaM–Fas-Pep1 complex whereas ionic interactions appear to contribute to the formation of the Ca^2+^/CaM–Fas-Pep2 complex.

### Characterization of the interaction interface of Ca^2+^/CaM with FasDD peptides by NMR spectroscopy

**Fig 5:**
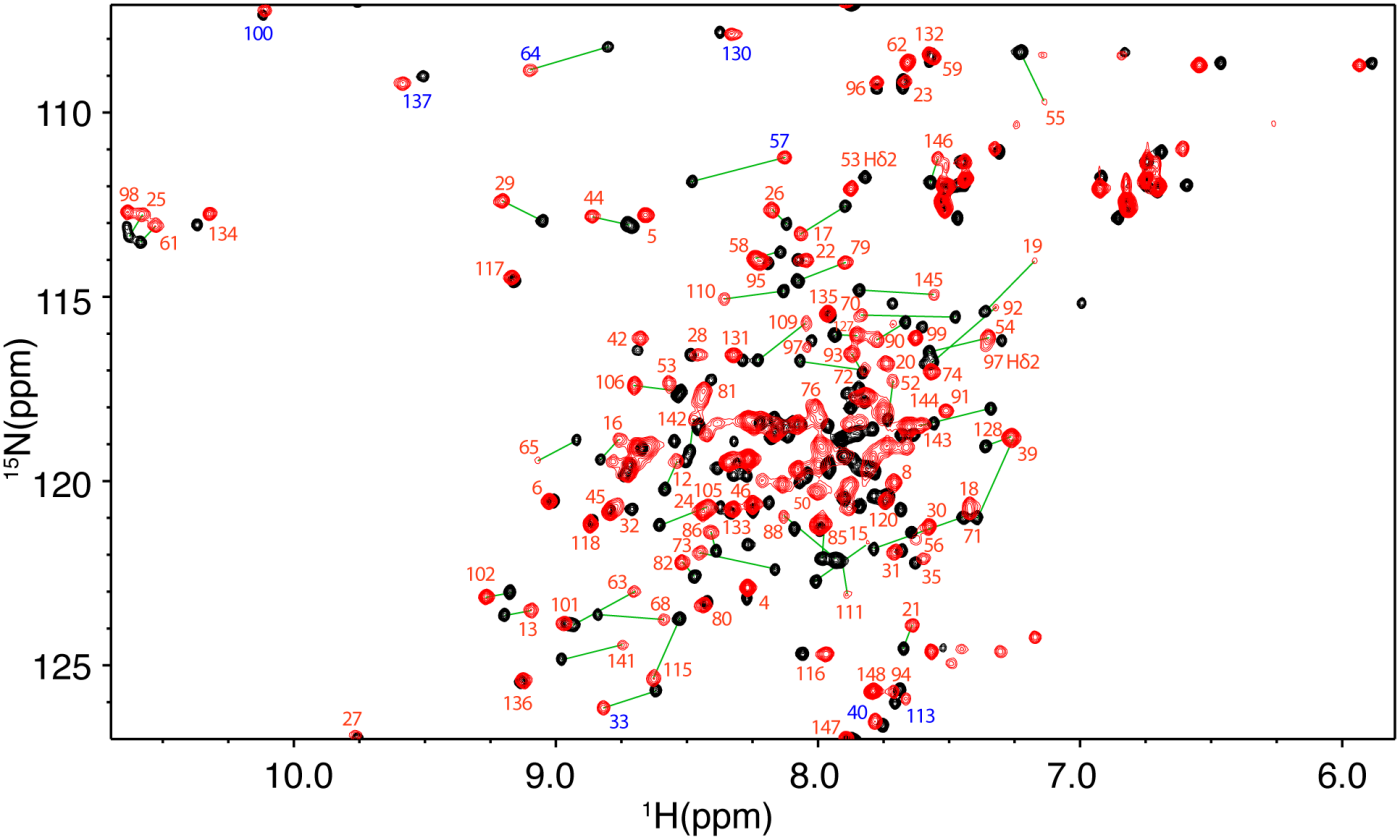
2D HSQC NMR data of Ca^2+^/CaM:Fas-Pep1 complex. Overlay of 2D ^1^H-^15^N HSQC spectra obtained for ^15^N-labeled Ca^2+^/CaM in the free state (black) and in complex with Fas-Pep1 (red) at 1.5:1 peptide: Ca^2+^/CaM. No chemical shift changes were observed in the HSQC spectra with further addition of Fas-Pep1, indicating saturation at this ratio. Signal labels correspond to residues of CaM in the bound form. Signals labeled in blue are folded in the spectrum by 20 ppm.

To gain insights into the molecular elements of Ca^2+^/CaM–FasDD interaction and to identify the interaction interface, we have utilized NMR chemical shift perturbation (CSP) as detected in ^1^H-^15^N heteronuclear single quantum coherence (HSQC) spectra. These experiments not only allow for identification of residues involved in binding but can also provide information on the induced conformational changes within proteins. 2D ^1^H-^15^N HSQC data obtained for a uniformly ^15^N-labeled Ca^2+^/CaM upon titration with unlabeled Fas-Pep1 are shown in Fig 5. Addition of substoichiometric amounts of Fas-Pep1 (0.5:1 peptide:Ca^2+^/CaM) led to a decrease in intensity for a significant number of ^1^H-^15^N resonances accompanied by appearance of several new signals, consistent with a slow exchange on the NMR scale between the free and bound forms of Ca^2+^/CaM. A steady decrease in intensity for the original ^1^H-^15^N signals and increase in intensity of the new signals was clearly observed with further addition of Fas-Pep1. Spectral changes ceased upon completion of peptide titration at 1.5:1 Fas-Pep1:Ca^2+^/CaM ratio when only the new set of signals was present (Fig 5). The vast majority of ^1^H-^15^N resonances of Ca^2+^/CaM exhibited chemical shift changes. The most significant chemical shift changes (Δδ > 0.2 ppm) were observed for signals corresponding to residues A15, F19, G33, G54, V55, A57, I63, D64, F68, L69, T70, M71, M72, A73, K77, D78, T79, E84, I85, A88, F92, L105, H107, M109, T110, G113, K115, V121, I125, F141, V142, Q143, M144, and M145 (Fig 5).

**Fig 6:**
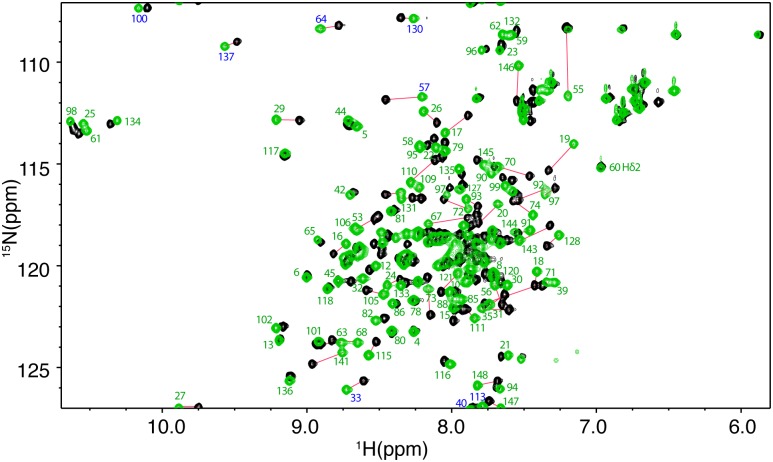
2D HSQC NMR data for Ca^2+^/CaM:Fas-Pep2 complex. Overlay of 2D ^1^H-^15^N HSQC spectra obtained for ^15^N-labeled Ca^2+^/CaM in the free state (black) and in complex with Fas-Pep2 (green) at 1.5:1 peptide:CaM ratio. No chemical shift changes were observed in the HSQC spectra with further addition of Fas-Pep2, indicating saturation at this ratio. Signal labels correspond to residues of CaM in the bound form. Signals labeled in blue are folded in the spectrum by 20 ppm.

Next, we conducted 2D NMR titrations on a ^15^N-labeled Ca^2+^/CaM as a function of added Fas-Pep2. At 0.5:1 peptide:Ca^2+^/CaM, numerous ^1^H-^15^N resonances decreased in intensity while several new signals appeared, consistent with a slow exchange on the NMR scale between the free and bound forms of Ca^2+^/CaM. A steady decrease in intensity for the original ^1^H-^15^N signals and increase in intensity of the new signals was clearly observed with further addition of Fas-Pep2. Spectral changes ceased at 1.5:1 Fas-Pep2:Ca^2+^/CaM. Among the numerous ^1^H-^15^N resonances that exhibited substantial chemical shift changes (Δδ > 0.2 ppm) are those corresponding to residues S17, F19, D20, V55, A57, E67, L69, T70, M71, M72, A73, K75, F92, H107, T110, V121, A128, F141, Q143, M144, and T146 (Fig 6).

**Fig 7:**
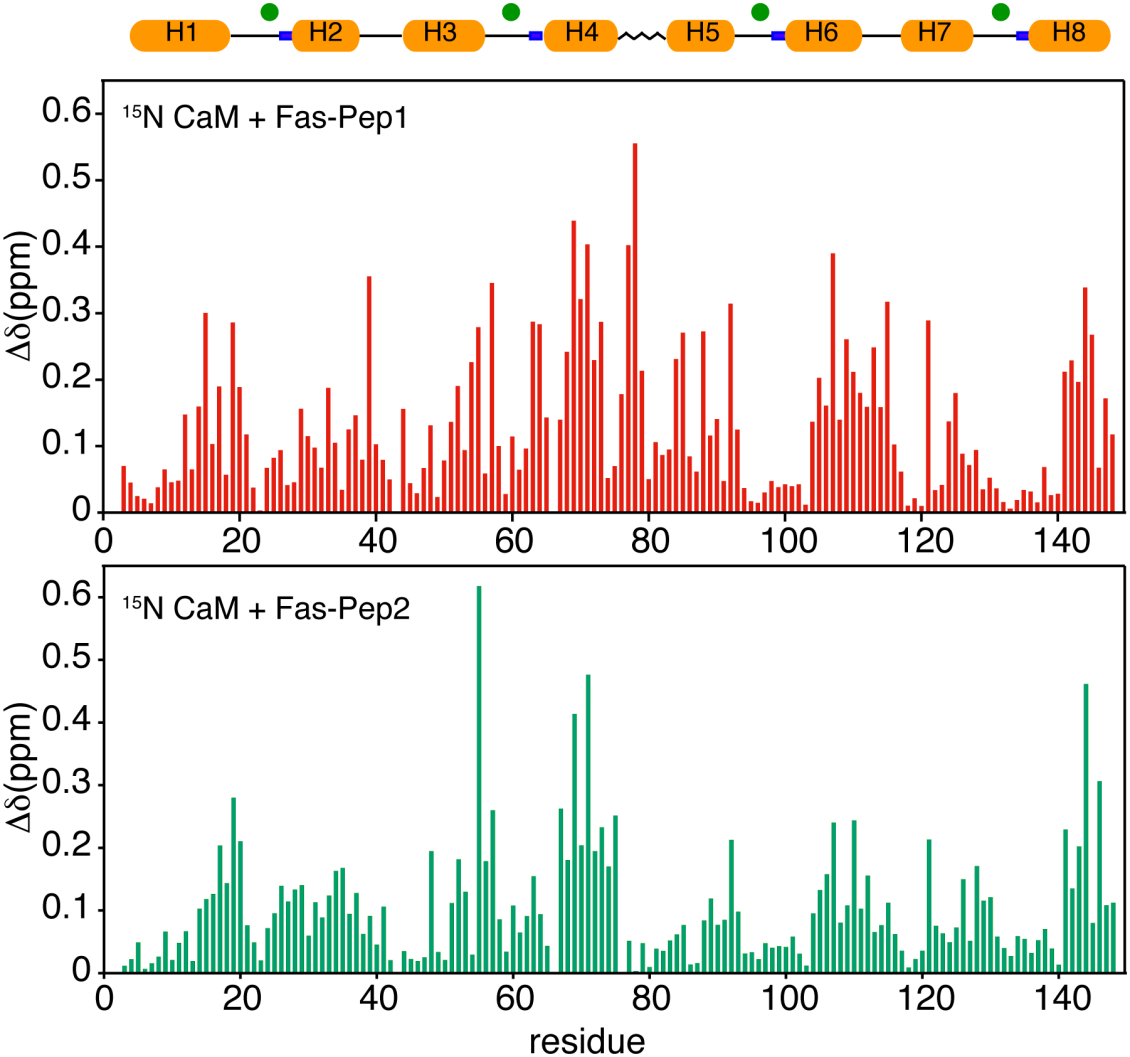
Histograms of the chemical shift changes of Ca^2+^/CaM upon binding to FasDD peptides. A schematic representation of the secondary structure of Ca^2+^/CaM is shown. Calcium ions are indicated by green dots. Histograms of normalized chemical shift changes vs. residue number for Ca^2+^/CaM bound to Fas-Pep1 and Fas-Pep2. Notice the differences in the chemical shift changes especially in the central linker (H4/H5) of Ca^2+^/CaM, which may suggest significant differences in the binding mode of FasDD peptides.

To assess whether there are major differences in the binding mode of FasDD peptides to Ca^2+^/CaM, we plotted the normalized maximum chemical shift changes vs. Ca^2+^/CaM residue number (Fig 7). Interestingly, the spectral changes induced by binding of FasDD peptides to Ca^2+^/CaM are significantly different. Overall, the magnitude of shift changes caused by Fas-Pep1 are larger than those observed upon Fas-Pep2 binding (Fig 7) and most of the differences in CSPs upon Fas-Pep2 binding are observed in the central region of Ca^2+^/CaM (residues 50–90). To visualize spatial distribution of residues affected by binding of the FasDD peptides, the CSPs were mapped on the Ca^2+^/CaM structure (S1 Fig). Residues perturbed by binding of Fas-Pep1 do not form a well-defined region but rather spread on both of the N- and C-terminal lobes and central linker, which suggests that Fas-Pep1 either engages a wide interface and/or induced a significant conformational change in the CaM protein. On the contrary, residues perturbed by binding of Fas-Pep2 are narrowly localized within the hydrophobic cores of the N- and C-terminal lobes but not in the central linker. Altogether, these results suggest some differences in the biding mode of FasDD peptides to Ca^2+^/CaM.

### FasDD peptides are α-helical when bound to Ca^2+^/CaM

Far-UV CD spectra of Ca^2+^/CaM, Fas-Pep1, Fas-Pep2 and their complexes have been obtained to determine whether formation of the complexes induce any major changes in the secondary structures of the peptides and/or Ca^2+^/CaM protein. The CD spectra of the free peptides display a negative band at ~200 nm consistent with a random coil, while that of the Ca^2+^/CaM protein shows two minima at 208 and 222 nm, consistent with an α-helical structure (S2 Fig). The CD spectra of the complexes are similar to those of Ca^2+^/CaM with features distinctive of an α-helical type. Relatively minor changes are observed in the CD signal of Ca^2+^/CaM upon binding to FasDD peptides. Only a small decrease is observed in the intensity of CD signal at 208 and 222 nm upon Fas-Pep1 binding to Ca^2+^/CaM, which indicates that the complex retains the α-helical character. An increase in the intensity of the CD signal at 208 and 222 nm upon binding of Fas-Pep2 to Ca^2+^/CaM suggests a small increase in the α-helical character of the complex. Induction of the α-helical character of Fas-Pep2 upon binding to Ca^2+^/CaM has been confirmed by NMR spectroscopy. The 2D ^1^H-^15^N HSQC spectrum obtained for a ^15^N-labeled Fas-Pep2 shows a narrow dispersion of the proton resonances, indicating a lack of ordered structure (S3 Fig). Upon binding of Ca^2+^/CaM, almost all ^1^H-^15^N signals of Fas-Pep2 exhibited chemical shift changes resulting in larger signal dispersion in the proton dimension. The backbone atom resonances of ^15^N and ^13^C-,^15^N-labeled Fas-Pep2 when bound to Ca^2+^/CaM were assigned using triple resonance NMR experiments (Materials and Methods). Numerous amide-amide cross signals are observed in the 3D ^15^N-edited HSQC-NOESY spectrum (S3 Fig), indicating that Fas-Pep2 adopts an α-helical conformation upon binding to CaM. The chemical shifts of CaM-bound Fas-Pep2 were also used to predict its order parameters and secondary structure content in TALOS+.[38] The chemical shift index (CSI) data indicate that Fas-Pep2 adopts an α-helical conformation upon binding to Ca^2+^/CaM (S4 Fig). These two α-helical motifs are similar to those observed in the unbound FasDD protein, which suggests that the α-helical motifs in Fas-Pep2 are retained within the complex.

### Is binding of FasDD peptides to Ca^2+^/CaM competitive or cooperative?

The above findings clearly indicate that two molecules of CaM bind to two distinct motifs on FasDD to form a ternary complex. The ITC data revealed that Fas-Pep1 binds slightly tighter (~4-times) to CaM than Fas-Pep2. We wanted to test whether binding of peptides is competitive or cooperative and whether the two peptides are able to bind simultaneously to the same molecule of CaM. To do so, we conducted 2D NMR titrations on a ^15^N-labeled Fas-Pep2 as a function of added unlabeled CaM and Fas-Pep1. First titration experiments were performed by addition of unlabeled CaM to a ^15^N-labeled sample of Fas-Pep2 (100 μM) at 1.5:1 CaM:peptide followed by acquisition of 2D ^1^H-^15^N HSQC data (S5 Fig). As expected, substantial chemical shift changes were observed for all ^1^H-^15^N resonances, indicating direct binding. Next, unlabeled Fas-Pep1 was added to the complex followed by acquisition of 2D HSQC spectrum (S5 Fig). All ^1^H and ^15^N signals shifted to positions similar to those observed for free Fas-Pep2, indicating that Fas-Pep1 displaced Fas-Pep2. Based on these findings, we conclude that binding is not cooperative and that the two FasDD peptides are not able to bind simultaneously to the same molecule of CaM.

### Binding of shorter Fas-Pep1 analogs to Ca^2+^/CaM

A recent study revealed that FasDD(214–238) binds to Ca^2+^/CaM much weaker (60–fold) than Fas-Pep1.[40] Only residues 214–227 have been detected in the x-ray structure of Ca^2+^/CaM bound to FasDD(214–238); no electron density has been detected for residues 228–238.[40] These findings are not in agreement with our current and previous findings.[32] To assess whether FasDD(214–238) binds to CaM in a manner similar to Fas-Pep1, we conducted NMR titration studies on FasDD(214–238). FasDD(214–238) was titrated into a ^15^N-labeled Ca^2+^/CaM followed by acquisition of 2D ^1^H-^15^N HSQC NMR data. Substantial CSPs are observed in the HSQC spectra (S6 Fig). Interestingly, the spectral changes are significantly different from those observed when Fas-Pep1 is bound to Ca^2+^/CaM, suggesting that the N-terminal residues of Fas-Pep1 (209–213) are probably involved in CaM binding. Next, we wanted to test whether FasDD(228–238) binds to Ca^2+^/CaM. This peptide (MTLSQVKGFVRKNGV) contains a classical 1-5-10 CaM-binding motif (underlined residues) and was predicted by a web-based tool as the CaM-binding domain. We obtained 2D HSQC NMR data on a ^15^N-labeled Ca^2+^/CaM sample as a function of added FasDD(228–238). Numerous ^1^H-^15^N signals exhibited significant CSPs in the HSQC spectra (S7 Fig), indicating direct binding. However, these shifts are substantially different from those observed for Fas-Pep1 and FasDD(214–238). Furthermore, as indicated by the chemical shift changes binding is in fast exchange on the NMR scale between the free and bound forms of Ca^2+^/CaM. Binding of FasDD(214–238) caused significant CSPs to signals corresponding to residues localized mainly in the C-terminus, indicating a preferential binding to the C-terminal lobe of Ca^2+^/CaM (S7 Fig). Altogether, our results indicate that residues 209–213 and 224–238 of FasDD are involved in CaM binding and that Fas-Pep1 represents the most relevant CaM-binding motif.

### N- and C-terminal domains of Ca^2+^/CaM are required for binding of FasDD peptides

We have recently shown that both N- and C-terminal lobes of Ca^2+^/CaM are important for binding of full-length FasDD. The NMR data shown above suggest that both lobes of Ca^2+^/CaM are important for binding of both Fas-Pep1 and Fas-Pep2. One possible model to explain these findings is that peptides anchor to both the N- and C-terminal lobes of Ca^2+^/CaM. Another possible scenario is that peptides bind only to one lobe of Ca^2+^/CaM but induce a conformational change in the protein. To discern these two models and gain more insights into the mode of binding, we have devised two approaches. In the first approach, we titrated Fas-Pep1 and Fas-Pep2 separately into ^15^N-labled samples of the isolated Ca^2+^/CaM-N and Ca^2+^/CaM-C domains followed by acquisition of 2D ^1^H-^15^N HSQC NMR data. A subset of ^1^H-^15^N signals exhibited significant chemical shift changes upon titration of Fas-Pep1 into Ca^2+^/CaM-N (S8 and S9 Figs). The most pronounced CSPs correspond to residues F12, K13, F16, D20, T34, L48, V55, D56, A57, E67, F68, L69, T70, M71, M72, and A73. Of note, the chemical shift changes in the HSQC spectra indicate fast exchange, on the NMR scale, between free and bound forms. Titration data were fit by a one-site binding model giving a *K*_d_ of ~268 μM (S10 Fig), a value that is ~10^3^-fold weaker than that obtained for full-length Ca^2+^/CaM, demonstrating that Ca^2+^/CaM-N is not sufficient for binding of Fas-Pep1.

The 2D HSQC titration data obtained on Ca^2+^/CaM-C as a function of added Fas-Pep1 also show significant CSPs for a subset of ^1^H-^15^N signals. In particular, the most dramatic chemical shift changes are observed for residues D80, I85, A88, F92, H107, M109, T110, L112, V121, I125, V143, M144, M145, and T146 (S8 and S9 Figs). The affinity of Fas-Pep1 binding to Ca^2+^/CaM-C (*K*_d_ = 76 μM, S10 Fig) is also much lower than that observed for full-length Ca^2+^/CaM. Altogether, these results show that Fas-Pep1 binds weakly to isolated lobes of Ca^2+^/CaM, suggesting that the peptide anchors simultaneously to both lobes. Similar experiments conducted on Fas-Pep2 also show that the peptide binds significantly weaker to Ca^2+^/CaM-N and Ca^2+^/CaM-C than to the full-length Ca^2+^/CaM protein (*K*_d_ ~ 100 and 15 μM, respectively; S10 Fig). Like Fas-Pep1, binding of Fas-Pep2 to Ca^2+^/CaM-N and Ca^2+^/CaM-C led to significant CSPs for numerous residues (S8 and S9 Figs). Our NMR titration data obtained on the isolated Ca^2+^/CaM-N and Ca^2+^/CaM-C suggest that neither domain alone is sufficient for strong peptide binding.

**Fig 8:**
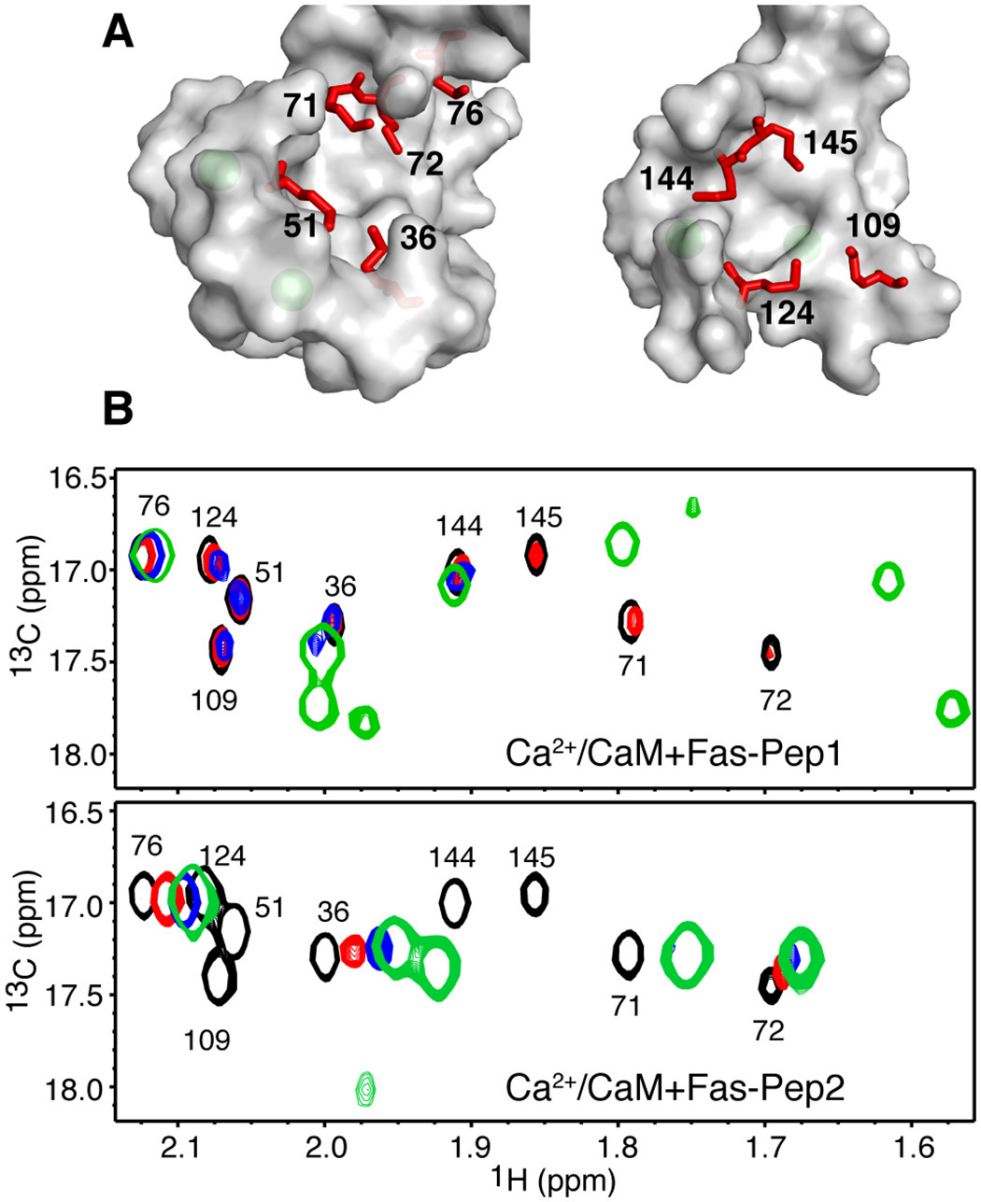
Role of Ca^2+^/CaM Met residues in binding of FasDD peptides. (**A**) Surface representation of the Ca^2+^/CaM structure (PDB ID: 3CLN) showing the nine Met residues localized in the N- and C-terminal lobes (red sticks). Ca^2+^ ions are colored in green. (**B**) Overlay of a selected region of 2D ^1^H-^13^C HMQC spectra obtained for ^13^C-labeled Ca^2+^/CaM as a function of added Fas-Pep1 (top) or Fas-Pep2 (bottom). [peptide:Ca^2+^/CaM = 0:1 (black), 0.5:1 (red), 1:1 (blue), 1.5:1 (green)]. Only the ^1^H-^13^C signals for the Met methyl groups are shown.

In the second approach, we assessed the role of hydrophobic surfaces located on the N- and C-terminal lobes of Ca^2+^/CaM. These hydrophobic surfaces, which contribute to the flexibility and function of Ca^2+^/CaM, are only formed when Ca^2+^ is bound.[24] Ca^2+^ binding induces a helical rearrangement, leading to exposure of eight methionine (Met) residues.[46, 47] Met residues are essential for the unique promiscuous binding behavior of Ca^2+^/CaM to target proteins.[47] The methyl groups of Met residues are useful “NMR reporters” and widely used to probe for target binding.[34, 42, 47–49] Five Met residues (36, 51, 71, 72 and 76) are located in the N-terminal lobe and four (109, 124, 144, and 145) are located within the hydrophobic patch in the C-terminal lobe (Fig 8A). To assess whether the N- and C-terminal hydrophobic surfaces contribute to the binding of FasDD peptides, we collected 2D ^1^H-^13^C HMQC data on a uniformly ^13^C-labeled Ca^2+^/CaM sample as titrated with Fas-Pep1 or Fas-Pep2. A selected region showing the ^1^H-^13^C signals of the methionine methyl groups (Cε) is shown in Fig 8B. Addition of a substoichiometric amount of Fas-Pep1 (0.5:1 peptide:Ca^2+^/CaM) led to a decrease in the intensity of ^1^H-^13^C signals of M51, M71, M72, M109, M124 and M145 (Fig 8B, upper panel). Minimal or no chemical shift changes have been observed for signals corresponding to M76 and M144. Further addition of Fas-Pep1 led to appearance of new signals (Fig 8B). At saturation (1.5:1 peptide:Ca^2+^/CaM), all nine ^1^H-^13^C signals are clearly observed. The HMQC NMR data indicate that Met residues from both the N- and C-terminal lobes contribute to Fas-Pep1 binding.

Notable CSPs are also observed in all of the ^1^H-^13^C resonances of Met residues upon binding of Fas-Pep2 to Ca^2+^/CaM (Fig 8B, lower panel). The chemical shift changes induced by binding of the two peptides are, however, different. For example, the most obvious differences are observed for signals corresponding to Met residues 71, 72, 144, and 145. These results suggest that not only both domains of Ca^2+^/CaM are important for binding of both peptides but also that the mode of binding is probably different. Related to this point, we analyzed the x-ray structure of Ca^2+^/CaM bound to FasDD(214–227) and noticed that the side chains of all Met residues (side chain of Met 76 is missing in the structure) are in close proximity to the peptide. Our data (Fig 8), however, show that the He signals of M76 and M144 do not exhibit chemical shift changes upon binding of Fas-Pep1, suggesting that these three Met residues are not perturbed by Fas-Pep1 binding. These results suggest that the binding mode of Fas-Pep1 to CaM is different from that described in the X-ray structure. Taken together, our NMR data indicate that both the N- and C-terminal lobes of Ca^2+^/CaM are involved in FasDD binding and that the hydrophobic surfaces formed by Met residues contribute to binding.

## Discussion

The interaction between Fas and FasL followed by binding of the FasDD to FADD DD triggers a cascade of caspase activation, leading to a proper execution of cell death.[2, 5–8, 50] Previous studies have shown that Ca^2+^/CaM is recruited into DISC in cholangiocarcinoma [10–16] and pancreatic cancer cells,[17] where it specifically interacts with FasDD, suggesting a novel regulatory role of CaM in Fas–mediated apoptosis.[11] Previous mutagenesis and CaM pull-down assays suggested that the CaM-binding site in FasDD is located between residues 214–254.[11] In a recent study,[32] we have shown that two molecules of Ca^2+^/CaM bind to one molecule of FasDD. The nature of the interaction and the size of the complex precluded full characterization of the interaction interface by NMR spectroscopy. Here, we employed biochemical, biophysical and NMR approaches to identify the Ca^2+^/CaM-binding domains of FasDD and determine the interaction interface. Our proteolytic digestion assays aided by mass spectrometry analysis revealed that the CaM-binding regions of FasDD span residues 209–239 and 251–288. Our data show that peptides containing these amino acids bind to Ca^2+^/CaM with a 1:1 stoichiometry. The binding affinities obtained for Fas-Pep1 and Fas-Pep2 are in agreement with the apparent affinity obtained for the intact FasDD protein. Our ITC data show that binding of Fas-Pep1 to Ca^2+^/CaM is entropically driven while that of Fas-Pep2 is enthalpically driven, indicating that a combination of electrostatic and hydrophobic forces contributes to the stabilization of the FasDD–Ca^2+^/CaM complex. Additionally, we have shown that the N- and C-terminal lobes of Ca^2+^/CaM are required for binding of both peptides. In particular, the hydrophobic surfaces formed by the Met residues on both lobes appear to be involved in binding. Whereas no previous studies have predicted the formation of a ternary Ca^2+^/CaM–FasDD complex, our recent [32] and current findings provide compelling evidence for the involvement of two distinct motifs of FasDD in binding to CaM.

The entropic factor in FasDD–CaM binding is likely important because CaM induces unfolding of FasDD, which greatly increases entropy. Therefore, it is not easy to discern the relative contributions of hydrophobic and electrostatic interactions in CaM binding to full-length FasDD. Binding of individual peptides is a complex process from the thermodynamic point of view because it is likely to be a multi-step process. If free peptides are disordered they first have to adopt an α-helical structure upon interacting with CaM, which decreases entropy of the system. The interaction with either peptide then may be entropically- or enthalpically-driven *per se*. The observed overall energetics is then a combination of folding and interaction and may, in fact, yield apparent thermodynamic parameters. It is an analogy of FasDD unfolding and binding by CaM.

CaM–protein complexes exhibit high variability in terms of overall structures. The two highly adaptable hydrophobic surfaces on the N- and C-terminal lobes of Ca^2+^/CaM, together with the flexible central linker, allow it to bind to numerous targets.[24, 27, 28] The Ca^2+^/CaM-binding regions of target proteins are typically short (15–20 residues), hydrophobic-basic in nature, and have the propensity to form an α-helix.[21, 27] While having all other characters, Fas-Pep1 and Fas-Pep2 are, however, significantly longer. In many of the classical Ca^2+^/CaM-binding targets, hydrophobic residues usually occupy positions at 1-5-10 or 1-8-14. Although these patterns are found in numerous CaM-binding proteins, other unclassified and rare examples have been observed.[21] Analysis of the Fas-Pep1 using this method yielded the highest score for residues 220–234, which contains a classical 1-5-10 motif (YITTIAGVMTLSQVKGFVR). Fas-Pep1 also contains a 1-8-14 motif (YITTIAGVMTLSQVKGFVR). The Ca^2+^/CaM protein adopts a collapsed conformation when bound to peptides with 1-5-10 or 1-8-14 motifs.[23, 28] As found in other systems,[34, 51] it is not that straightforward to predict the mode of binding of peptides to CaM based on sequences alone. Fas-Pep2 also contains a 1-5-10 motif (LIKDLKKANL) but has not been predicted to bind to Ca^2+^/CaM. Sequence dissimilarity of studied FasDD peptides may lead to different CaM-binding modes.

In addition to the collapsed model, several other models have been described. For example, a semi-extended modular architecture is formed upon binding of a long peptide (36 amino acids) derived from the human immunodeficiency virus type-1 matrix protein (HIV-1 MA).[51] In this model, the peptide interacts with CaM via two well-separated α-helical motifs. A similar bipartite binding motif has also been identified for CaM bound to Munc13-1, a regulator of synaptic vesicle priming [52]. Other non-canonical models have also been described. For example, (i) CaM simultaneously binds two peptides derived from plant glutamate decarboxylase, which induces enzyme dimerization.[53] (ii) Binding of the anthrax edema factor to Ca^2+^/CaM with four discrete regions of oedema factor form a surface that recognizes an extended conformation of CaM, which is very different from the collapsed conformation observed in other structures of CaM bound to effector peptides.[54] (iii) Binding of SK channels to CaM in which CaM interacts with three α-helices in an extended conformation.[55] (iv) Structure of a domain swapped hetero-tetramer of calcineurin where each peptide is bound by a N-lobe and a C-lobe of different CaM molecules, which are in extended conformations.[56] These examples suggest many possibilities, including one in which two molecules of CaM bind FasDD with their N-lobe and C-lobe engaged to non-contiguous sequence (e.g., bridging the Fas-Pep1 and Fas-Pep2 regions). The precise structural details of CaM binding to Fas-Pep1 and Fas-Pep2 remain to be elucidated.

We have previously proposed that FasDD becomes unfolded upon binding to Ca^2+^/CaM.[32] Our data described here also support unfolding of the FasDD tertiary structure. As shown in Fig 1, Fas-Pep1 and Fas-Pep2 are packed against each other and binding of these peptides to Ca^2+^/CaM likely requires their unpacking. The ability of Ca^2+^/CaM to disrupt the tertiary structure of protein targets is not unusual and has been observed in studies of the interactions between Ca^2+^/CaM and HIV-1 MA.[34, 42, 57] In other examples, partial unfolding of CaM targets is needed to activate enzymatically driven cleavage reactions.[58] Analysis of the FasDD and Ca^2+^/CaM protein structures indicates that the two proteins share significant structural features.[59] Structural studies have shown that upon binding to FADD, the FasDD protein undergoes a transition from a closed state to open state.[6] It was suggested that both proteins possess a “designed mobility” in which the intermolecular interactions in the complex disrupt the original structure, causing a structural transformation that leads to a signaling event frequently associated with a hinge motion.[59] On the other hand, in most of the published structures of Ca^2+^/CaM complexes with target proteins/peptides the Ca^2+^/CaM protein undergoes a conformational change involving bending of the central linker to form a closed form.[20, 21] Exceptions, however, also exist. In a very few cases, Ca^2+^/CaM complexes adopt a modular architecture by which two α-helices of target protein, separated by a linker, anchor into the N and C-terminal lobes of Ca^2+^/CaM.[51, 52, 60] These findings led us to propose a model by which Ca^2+^/CaM induces a conformational switch that leads to opening of FasDD and engaging of Fas-Pep1 and Fas-Pep2, thus masking the FADD-interacting region. Indeed, the x-ray structure of FasDD with FADD DD shows that residues 209–233 and 270–310 of FasDD are involved in extensive intermolecular contacts with FADD DD.[6] Binding of Ca^2+^/CaM to these regions will greatly hinder its ability to bind to FADD, thus inhibiting the initiation of apoptotic signaling pathway.

Cholangiocarcinoma is the second most common primary malignant tumor of the liver and comprises approximately 20% of all hepatobiliary malignancies in the United States. A marked increase in the incidence and mortality from cholangiocarcinoma over the last two decades necessitates an effective search for a therapy regimen. Ca^2+^/CaM antagonists possess anti-proliferative activity [61] as they inhibit tumor cell invasion *in vitro* [62] and metastasis *in vivo*.[63] We have recently shown that Ca^2+^/CaM antagonists inhibit its binding to FasDD, providing a molecular basis for their role in inducing Fas–mediated apoptosis in cholangiocarcinoma cells.[10, 14, 18] CaM can also affect other pathways in liver tumor cholangiocarcinoma. For instance, the RAS pathway is also coupled with the Ca^2+^/CaM pathway.[64] Molecular characterization of signaling pathways involving Fas is critical for identifying new targets that are crucial in switching between the death vs. survival signals in response to the same ligand.

## Supporting Information

S1 Fig Mapping of CSPs on the Ca^2+^/CaM structure. Cartoon representation of Ca^2+^/CaM structure (PDB ID: 1CLL) colored according to the magnitude of ^1^H-^15^N chemical shift changes (blue: minimal, red: maximal) induced by binding of Fas-Pep1 (top) and Fas-Pep2 (bottom). White spheres indicate Ca^2+^ atoms.
(PDF)

S2 Fig CD spectra of Ca^2+^/CaM complexes with FasDD peptides. Far-UV CD spectra obtained for FasDD peptides, Ca^2+^/CaM and their complexes. The CD spectra of the free peptides display a negative band at ~200 nm consistent with a random coil whereas that of the Ca^2+^/CaM protein shows two minima at 208 and 222 nm, consistent with an α-helical structure. The CD spectra of the complexes are similar to those of Ca^2+^/CaM with features distinctive of an α-helical type.
(PDF)

S3 Fig 2D HSQC and 3D NOESY-HSQC NMR spectra of Ca^2+^/CaM–Fas-Pep2. (**A**) Overlay of 2D ^1^H-^15^N HSQC spectra obtained for a ^15^N-labeled Fas-Pep2 in the free state (black) and in complex with Ca^2+^/CaM (red). Assignments for Ca^2+^/CaM-bound Fas-Pep2 are shown. The amide signal of residue 287 is folded in (actual ^15^N chemical shift = 127.3 ppm). (**B**) A selected slice of the three-dimensional ^15^N-edited HSQC-NOESY spectrum obtained for a ^15^N-labeled Fas-Pep2 in complex with unlabeled Ca^2+^/CaM. Several amide-amide cross speaks have been observed, indicating that Fas-Pep2 adopts an α-helical conformation within the complex. Assignments of NOE cross-peaks are indicated in black for the direct dimension and in red for the indirect dimension.
(PDF)

S4 Fig TALOS+ secondary structure prediction for Fas-Pep2 in complex with Ca^2+^/CaM. (**A**) Random coil index (RCI)-derived order parameters and (**B**) the probability of secondary structure (positive values are obtained for extended structure, negative for α-helix) plotted for Fas-Pep2 residues. Only secondary structure probabilities |SS| > 0.5 are shown.
(PDF)

S5 Fig NMR competition experiment. Overlay of 2D ^1^H-^15^N HSQC spectra obtained for a ^15^N-labeled Fas-Pep2 sample (100 μM) in the free state (black) and when bound to unlabeled Ca^2+^/CaM (red) at 1:1.5 (peptide:Ca^2+^/CaM). Fas-Pep1 was added to the CaM:Fas-Pep2 sample at followed by acquisition of 2D ^1^H-^15^N HSQC (green). As shown, the ^1^H-^15^N signals of Fas-Pep2 reverted back close to the positions observed for free Fas-Pep2, indicating that Fas-Pep2 is displaced by Fas-Pep1.
(PDF)

S6 Fig NMR titration of FasDD(214–238) into Ca^2+^/CaM. Overlay of 2D ^1^H-^15^N HSQC spectra obtained for a ^15^N-labeled Ca^2+^/CaM sample (100 μM) in the free state (black), when bound to Fas-Pep1 at 1.5:1 peptide:Ca^2+^/CaM (red), and when bound to a FasDD(214–238) at 2:1 peptide:Ca^2+^/CaM (green). Notice that the chemical shift perturbations induced by Fas-Pep1 are significantly different from those induced by FasDD(214–238), suggesting that the binding mode of the two peptides to Ca^2+^/CaM is different.
(PDF)

S7 Fig NMR titration data. Overlay of 2D ^1^H-^15^N HSQC spectra obtained for a ^15^N-labeled Ca^2+^/CaM sample (100 μM) upon binding to FasDD(224–238). Interestingly, as indicated by the chemical shift perturbations the peptide appears to bind to the C-terminal domain of Ca^2+^/CaM.
(PDF)

S8 Fig 2D HSQC NMR spectra of Ca^2+^/CaM-N and Ca^2+^/CaM-C titrated with Fas peptides. Overlay of 2D ^1^H-^15^N HSQC spectra obtained for ^15^N-labeled Ca^2+^/CaM-N and Ca^2+^/CaM-C samples (150 μM) upon titration with Fas-Pep1 or Fas-Pep2. As indicated by the fast exchange on the NMR scale between free and bound states, FasDD peptides bind weaker to the isolated N and C lobes when compared to the intact Ca^2+^/CaM protein.
(PDF)

S9 Fig Histograms of the chemical shift changes of Ca^2+^/CaM-N and Ca^2+^/CaM-C bound to Fas peptides. Histograms of normalized ^1^H-^15^N chemical shift changes vs. residue number calculated from the HSQC spectra for Ca^2+^/CaM-N and Ca^2+^/CaM-C complexes with Fas-Pep1 and Fas-Pep2. Notice that significant differences in chemical shift changes are observed upon binding of Ca^2+^/CaM-N or Ca^2+^/CaM-C to both peptides. For example, signals corresponding to the N-terminal residues of Ca^2+^/CaM-N (first 15 amino acids) exhibited substantial chemical shift changes upon binding of Fas-Pep1 (panel **A**). However, the ^1^H-^15^N signals corresponding to these residues were less sensitive to binding of Fas-Pep2 (panel **B**). Likewise, significant differences also exist in Ca^2+^/CaM-C residues perturbed upon binding of Fas-Pep1 vs. Fas-Pep2. Altogether, these results suggest that Fas-Pep1 and Fas-Pep2 bind to both of Ca^2+^/CaM-N and Ca^2+^/CaM-C, and that the binding mode of these peptides may be different.
(PDF)

S10 Fig Binding isotherms of Ca^2+^/CaM-N and Ca^2+^/CaM-C bound to Fas peptides. Binding isotherms generated by plotting the change in ^1^H and ^15^N chemical shifts (Δδ) as a function of peptide concentration. Titration data were fit by a one-site binding model. As indicated by the *K*_d_ values, FasDD peptides bind much weaker to isolated domains of Ca^2+^/CaM than that of the full-length protein (15–268 vs. 0.3 and 1.1 μM). Data also show that both peptides have higher affinity to Ca^2+^/CaM-C than Ca^2+^/CaM-N.
(PDF)

S1 Table Fas peptides identified by mass spectrometry. FasDD peptides obtained by subtilisin digestion and identified by mass spectrometry.
(PDF)
