# Supplementary material for: Identification of the Calmodulin-Binding Domains of Fas Death Receptor
Source: PLoS One. 2016 Jan 6;11(1):e0146493. doi: 10.1371/journal.pone.0146493 (PMC4703387; doi:10.1371/journal.pone.0146493)
Supplement: S1 Fig — Cartoon representation of Ca2+/CaM structure (PDB ID: 1CLL) colored according to the magnitude of 1H-15N chemical shift changes (blue: minimal, red: maximal) induced by binding of Fas-Pep1 (top) and Fas-Pep2 (bottom). White spheres indicate Ca2+ atoms. (PDF) [file pone.0146493.s001.pdf]

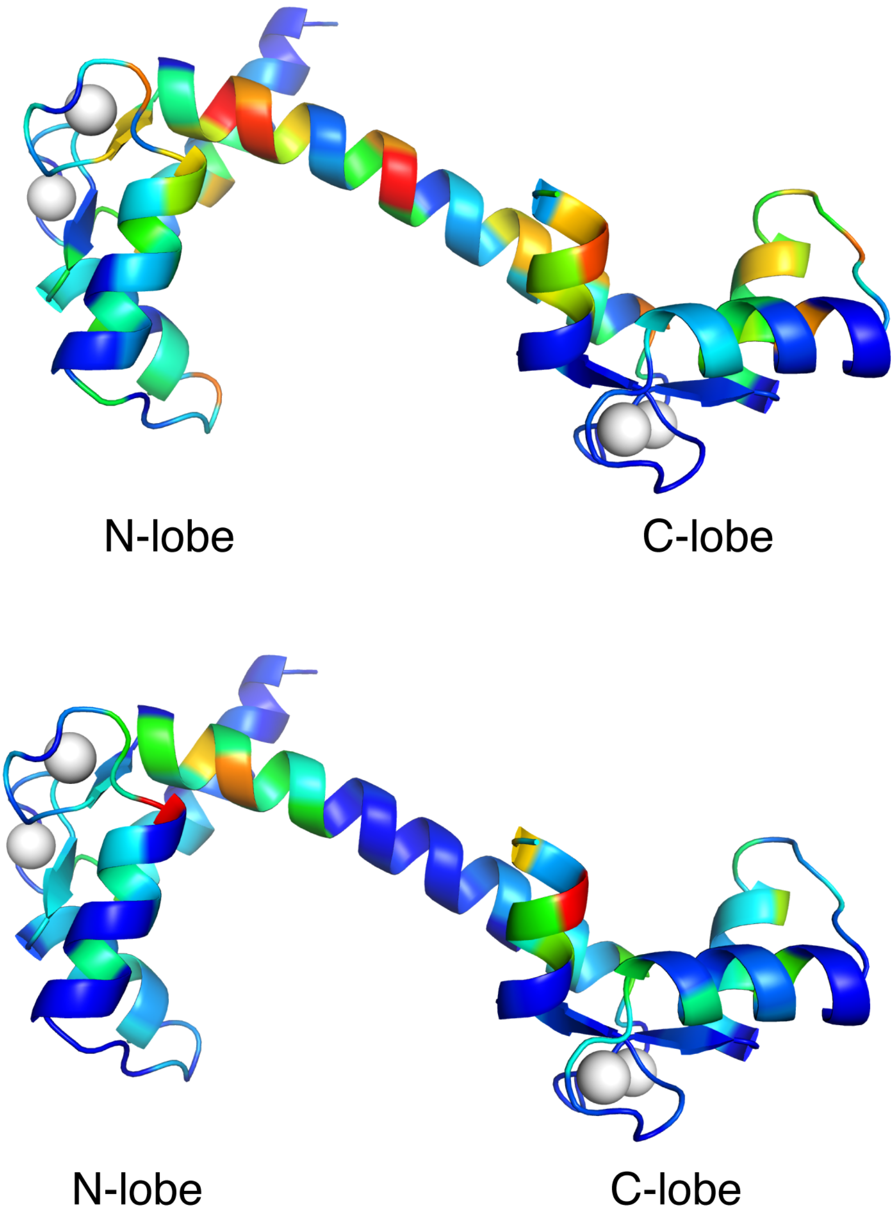

Figure S1. Cartoon representation of Ca^{2+}/CaM structure (PDB ID: 1CLL) colored according to the magnitude of ^{1}H-^{15}N chemical shift changes (blue: minimal, red: maximal) induced by binding of Fas-Pep1 (top) and Fas-Pep2 (bottom). White spheres indicate Ca^{2+} atoms.
