# Supplementary material for: Identification of the Calmodulin-Binding Domains of Fas Death Receptor
Source: PLoS One. 2016 Jan 6;11(1):e0146493. doi: 10.1371/journal.pone.0146493 (PMC4703387; doi:10.1371/journal.pone.0146493)
Supplement: S2 Fig — Far-UV CD spectra obtained for FasDD peptides, Ca2+/CaM and their complexes. The CD spectra of the free peptides display a negative band at ~200 nm consistent with a random coil whereas that of the Ca2+/CaM protein shows two minima at 208 and 222 nm, consistent with an α-helical structure. The CD spectra of the complexes are similar to those of Ca2+/CaM with features distinctive of an α-helical type. (PDF) [file pone.0146493.s002.pdf]

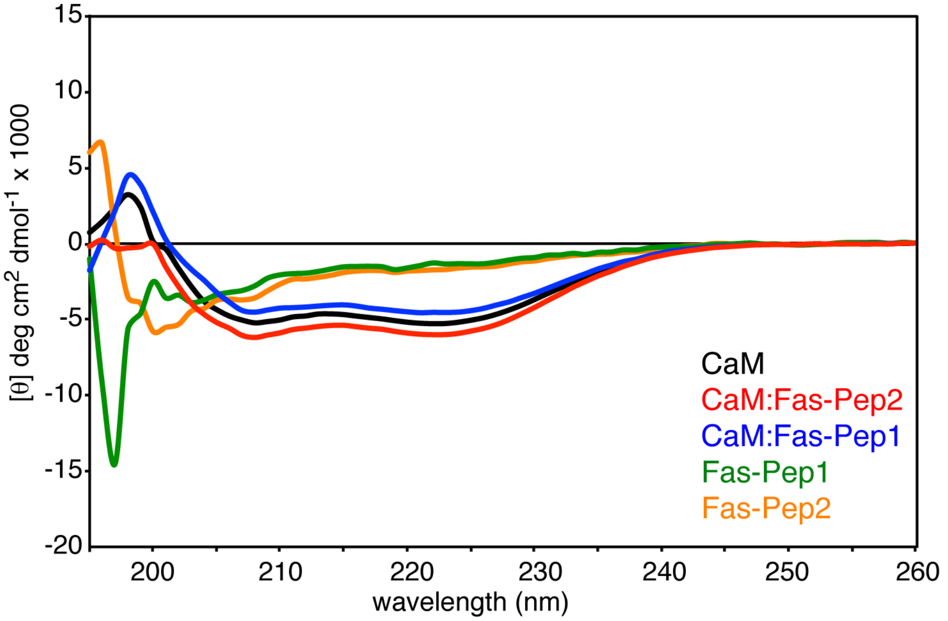

Figure S2. Far-UV CD spectra obtained for FasDD peptides, Ca^{2+}/CaM and their complexes. The CD spectra of the free peptides display a negative band at ~200 nm consistent with a random coil whereas that of the Ca^{2+}/CaM protein shows two minima at 208 and 222 nm, consistent with an α-helical structure. The CD spectra of the complexes are similar to those of Ca^{2+}/CaM with features distinctive of an α -helical type.
