# Supplementary material for: Identification of the Calmodulin-Binding Domains of Fas Death Receptor
Source: PLoS One. 2016 Jan 6;11(1):e0146493. doi: 10.1371/journal.pone.0146493 (PMC4703387; doi:10.1371/journal.pone.0146493)
Supplement: S3 Fig — (A) Overlay of 2D 1H-15N HSQC spectra obtained for a 15N-labeled Fas-Pep2 in the free state (black) and in complex with Ca2+/CaM (red). Assignments for Ca2+/CaM-bound Fas-Pep2 are shown. The amide signal of residue 287 is folded in (actual 15N chemical shift = 127.3 ppm). (B) A selected slice of the three-dimensional 15N-edited HSQC-NOESY spectrum obtained for a 15N-labeled Fas-Pep2 in complex with unlabeled Ca2+/CaM. Several amide-amide cross speaks have been observed, indicating that Fas-Pep2 adopts an α-helical conformation within the complex. Assignments of NOE cross-peaks are indicated in black for the direct dimension and in red for the indirect dimension. (PDF) [file pone.0146493.s003.pdf]

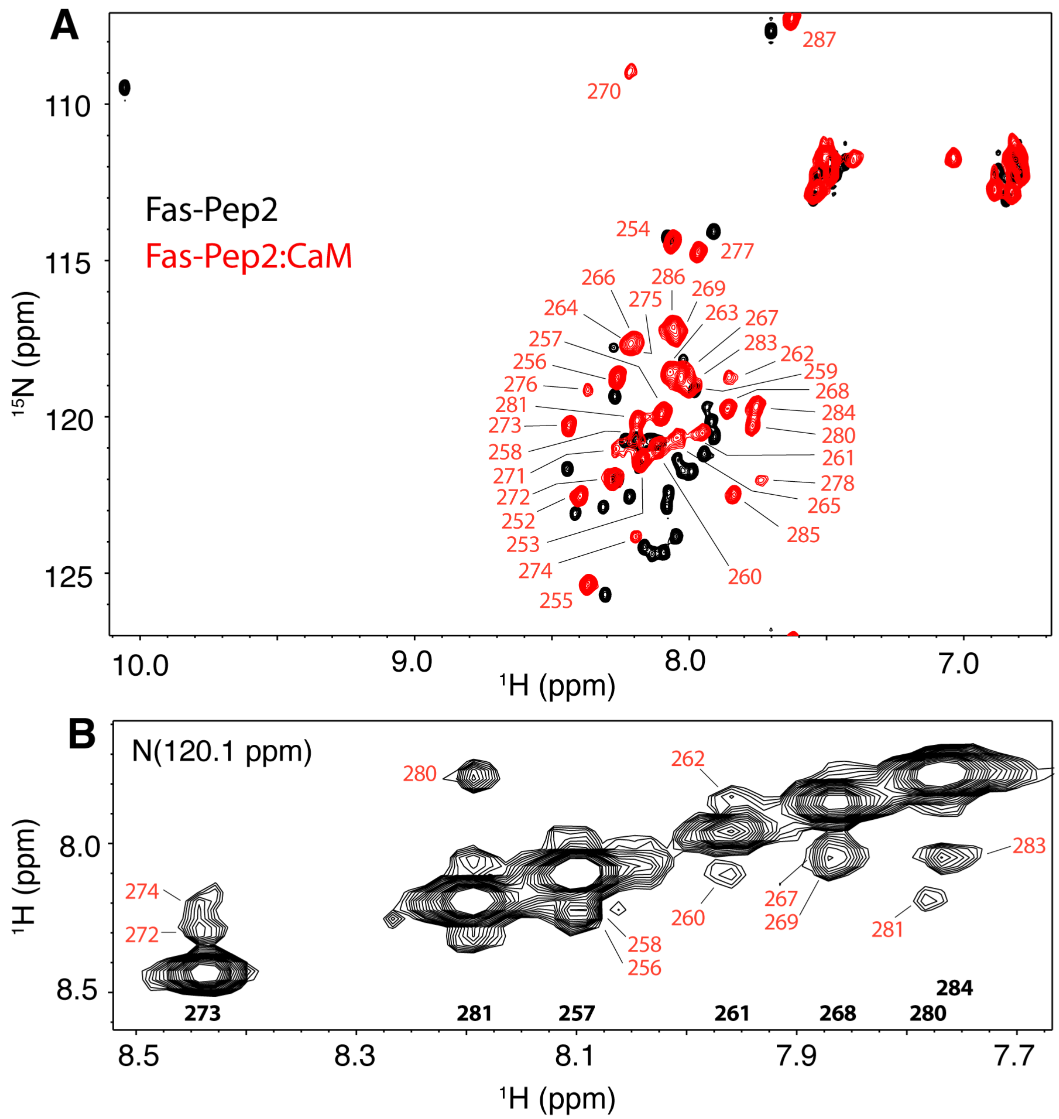

Figure S3. (**A**) Overlay of 2D ^{1}H-^{15}N HSQC spectra obtained for a ^{15}N-labeled Fas-Pep2 in the free state (black) and in complex with Ca^{2+}/CaM (red). Assignments for Ca^{2+}/CaM-bound Fas-Pep2 are shown. The amide signal of residue 287 is folded in (actual ^{15}N chemical shift = 127.3 ppm). (**B**) A selected slice of the three-dimensional ^{15}N-edited NOESY-HSQC spectrum obtained for a ^{15}N-labeled Fas-Pep2 in complex with unlabeled Ca^{2+}/CaM. Several amide-amide cross speaks have been observed, indicating that Fas-Pep2 adopts a helical conformation within the complex. Assignments of NOE cross-peaks are indicated in black for the direct dimension and in red for the indirect dimension.
