# Supplementary material for: Identification of the Calmodulin-Binding Domains of Fas Death Receptor
Source: PLoS One. 2016 Jan 6;11(1):e0146493. doi: 10.1371/journal.pone.0146493 (PMC4703387; doi:10.1371/journal.pone.0146493)
Supplement: S4 Fig — (A) Random coil index (RCI)-derived order parameters and (B) the probability of secondary structure (positive values are obtained for extended structure, negative for α-helix) plotted for Fas-Pep2 residues. Only secondary structure probabilities |SS| > 0.5 are shown. (PDF) [file pone.0146493.s004.pdf]

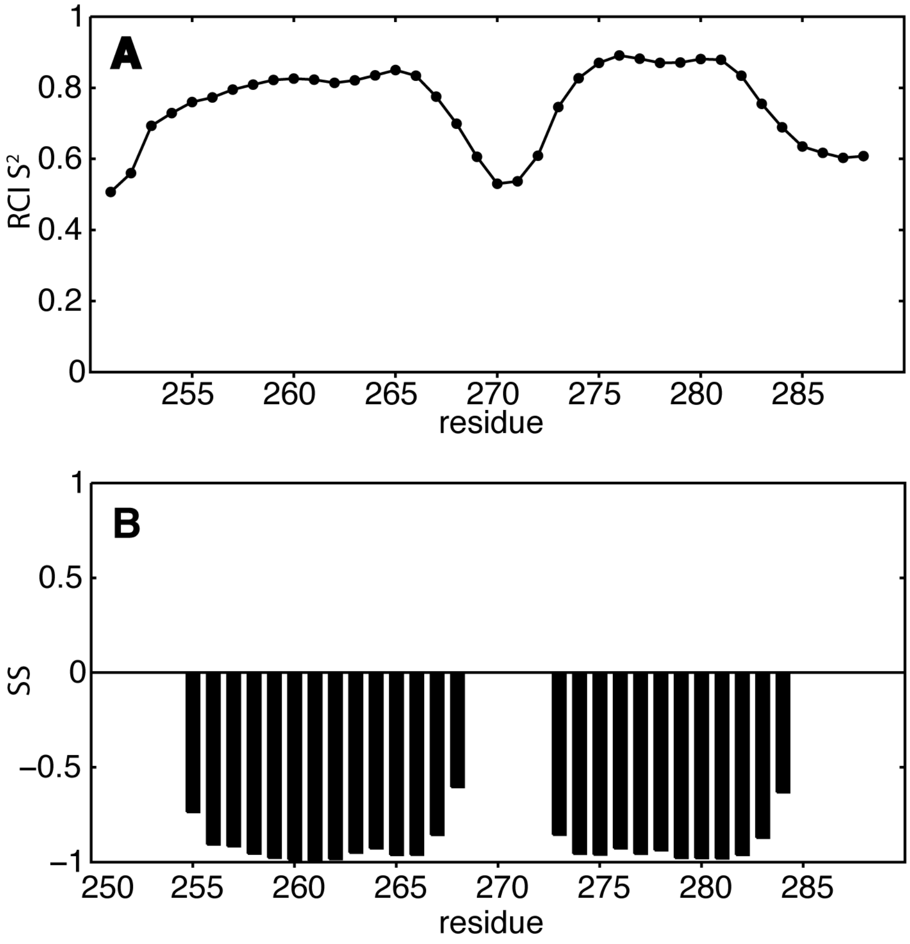

Fig S4. TALOS+ secondary structure prediction for Fas-Pep2 in complex with Ca^{2+}/CaM. (**A**) Random coil index (RCI)-derived order parameters and (**B**) the probability of secondary structure (positive values are obtained for extended structure, negative for α-helix) plotted for Fas-Pep2 residues. Only secondary structure probabilities |SS| > 0.5 are shown.
