# Supplementary material for: Identification of the Calmodulin-Binding Domains of Fas Death Receptor
Source: PLoS One. 2016 Jan 6;11(1):e0146493. doi: 10.1371/journal.pone.0146493 (PMC4703387; doi:10.1371/journal.pone.0146493)
Supplement: S5 Fig — Overlay of 2D 1H-15N HSQC spectra obtained for a 15N-labeled Fas-Pep2 sample (100 μM) in the free state (black) and when bound to unlabeled Ca2+/CaM (red) at 1:1.5 (peptide:Ca2+/CaM). Fas-Pep1 was added to the CaM:Fas-Pep2 sample at followed by acquisition of 2D 1H-15N HSQC (green). As shown, the 1H-15N signals of Fas-Pep2 reverted back close to the positions observed for free Fas-Pep2, indicating that Fas-Pep2 is displaced by Fas-Pep1. (PDF) [file pone.0146493.s005.pdf]

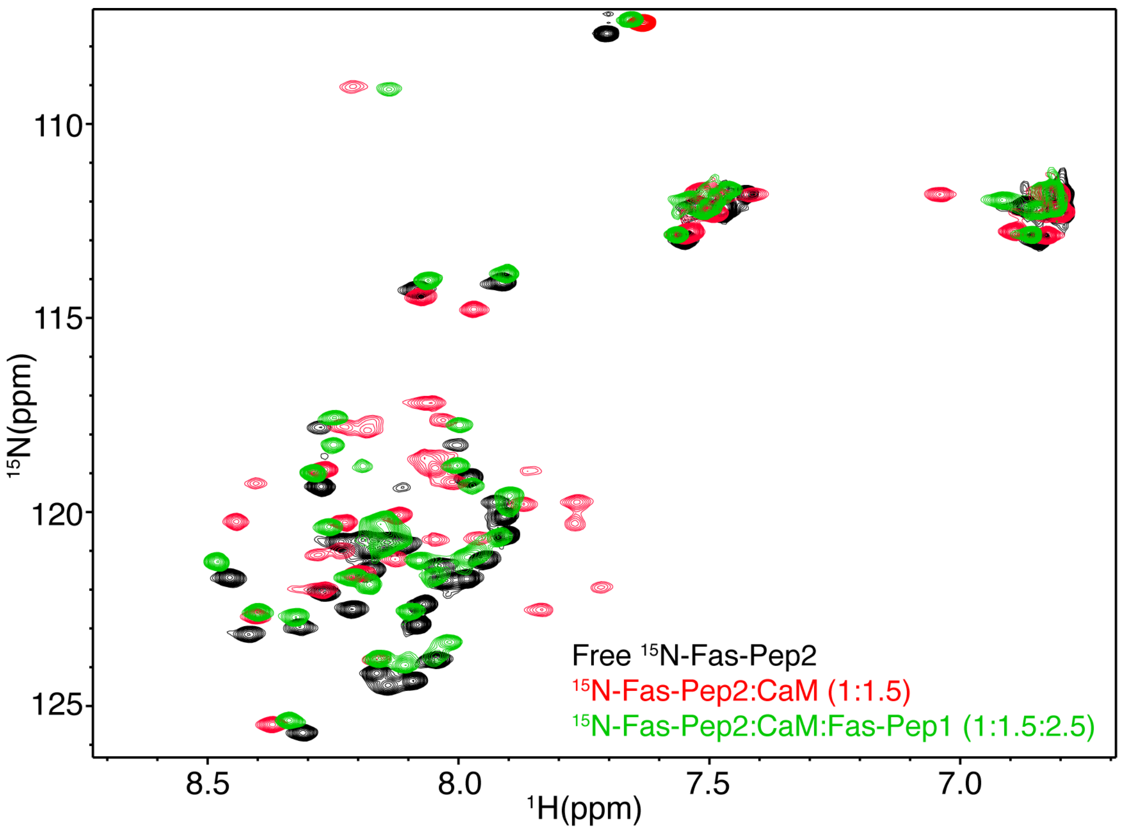

Fig S5. Overlay of 2D ^{1}H-^{15}N HSQC spectra obtained for a ^{15}N-labeled Fas-Pep2 sample (100 μM) in the free state (black) and when bound to unlabeled Ca^{2+}/CaM (red) at 1:1.5 (peptide:Ca^{2+}/CaM). Fas-Pep1 was added to the CaM:Fas-Pep2 sample at followed by acquisition of 2D ^{1}H-^{15}N HSQC (green). As shown, the ^{1}H-^{15}N signals of Fas-Pep2 reverted back close to the positions observed for free Fas-Pep2, indicating that Fas-Pep2 is displaced by Fas-Pep1.
