# Supplementary material for: Identification of the Calmodulin-Binding Domains of Fas Death Receptor
Source: PLoS One. 2016 Jan 6;11(1):e0146493. doi: 10.1371/journal.pone.0146493 (PMC4703387; doi:10.1371/journal.pone.0146493)
Supplement: S6 Fig — Overlay of 2D 1H-15N HSQC spectra obtained for a 15N-labeled Ca2+/CaM sample (100 μM) in the free state (black), when bound to Fas-Pep1 at 1.5:1 peptide:Ca2+/CaM (red), and when bound to a FasDD(214–238) at 2:1 peptide:Ca2+/CaM (green). Notice that the chemical shift perturbations induced by Fas-Pep1 are significantly different from those induced by FasDD(214–238), suggesting that the binding mode of the two peptides to Ca2+/CaM is different. (PDF) [file pone.0146493.s006.pdf]

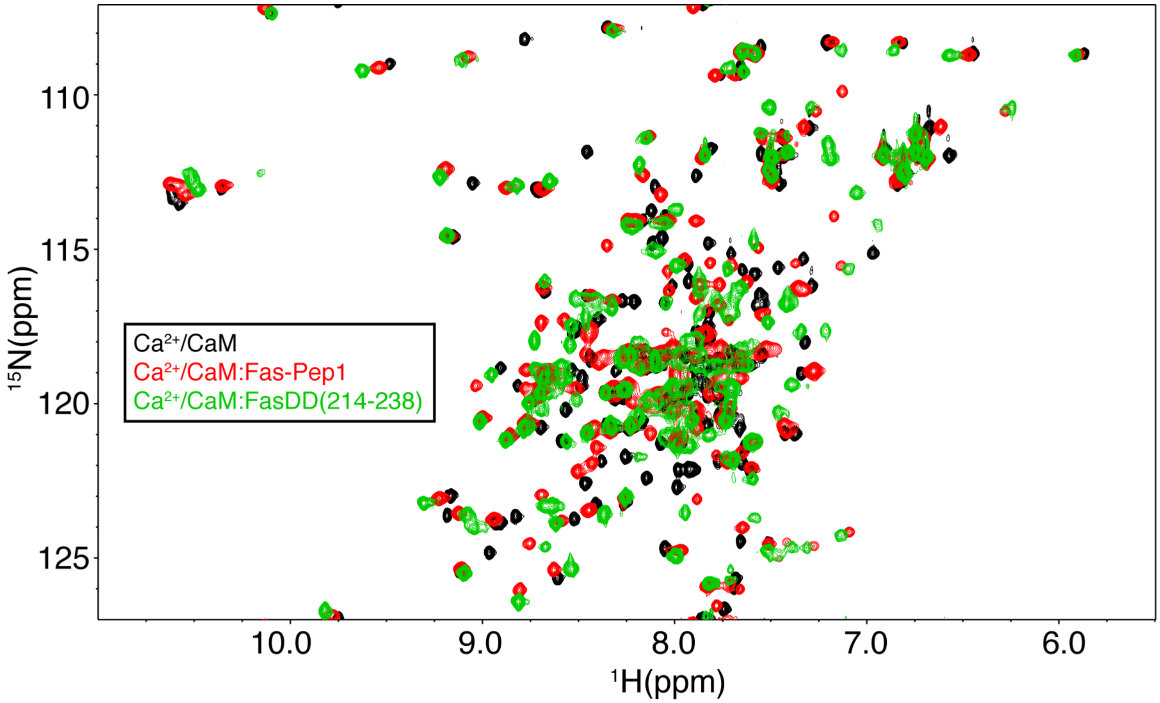

Fig S6. Overlay of 2D ^{1}H-^{15}N HSQC spectra obtained for a ^{15}N-labeled Ca^{2+}/CaM sample (100 μM) in the free state (black), when bound to Fas-Pep1 at 1.5:1 peptide:Ca^{2+}/CaM (red), and when bound to a FasDD(214-238) at 2:1 peptide:Ca^{2+}/CaM (green). Notice that the chemical shift perturbations induced by Fas-Pep1 are significantly different from those induced by FasDD(214-238), suggesting that the binding mode of the two peptides to Ca^{2+}/CaM is different.
