# Supplementary material for: Identification of the Calmodulin-Binding Domains of Fas Death Receptor
Source: PLoS One. 2016 Jan 6;11(1):e0146493. doi: 10.1371/journal.pone.0146493 (PMC4703387; doi:10.1371/journal.pone.0146493)
Supplement: S7 Fig — Overlay of 2D 1H-15N HSQC spectra obtained for a 15N-labeled Ca2+/CaM sample (100 μM) upon binding to FasDD(224–238). Interestingly, as indicated by the chemical shift perturbations the peptide appears to bind to the C-terminal domain of Ca2+/CaM. (PDF) [file pone.0146493.s007.pdf]

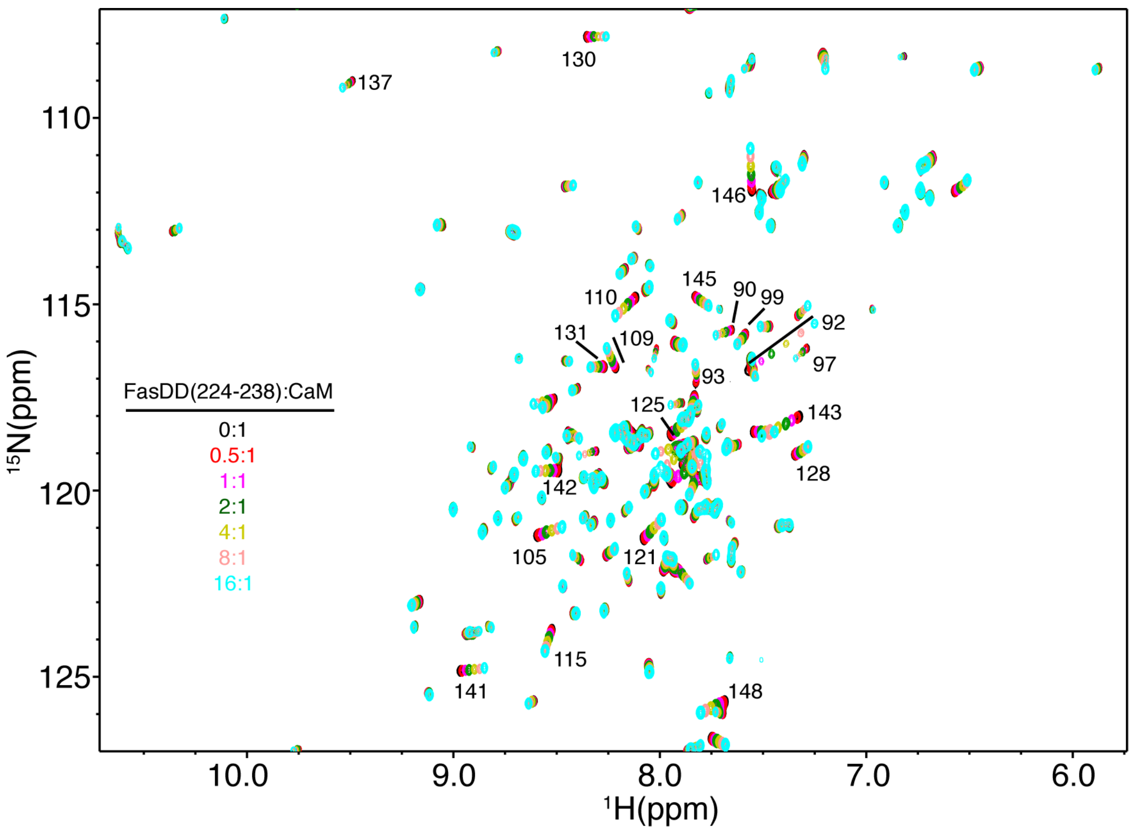

Fig S7. Overlay of 2D ^{1}H-^{15}N HSQC spectra obtained for a ^{15}N-labeled Ca^{2+}/CaM sample (100 μM) upon binding to FasDD(224-238). Interestingly, as indicated by the chemical shift perturbations the peptide appears to bind to the C-terminal domain of Ca^{2+}/CaM.
