# Supplementary material for: Identification of the Calmodulin-Binding Domains of Fas Death Receptor
Source: PLoS One. 2016 Jan 6;11(1):e0146493. doi: 10.1371/journal.pone.0146493 (PMC4703387; doi:10.1371/journal.pone.0146493)
Supplement: S8 Fig — Overlay of 2D 1H-15N HSQC spectra obtained for 15N-labeled Ca2+/CaM-N and Ca2+/CaM-C samples (150 μM) upon titration with Fas-Pep1 or Fas-Pep2. As indicated by the fast exchange on the NMR scale between free and bound states, FasDD peptides bind weaker to the isolated N and C lobes when compared to the intact Ca2+/CaM protein. (PDF) [file pone.0146493.s008.pdf]

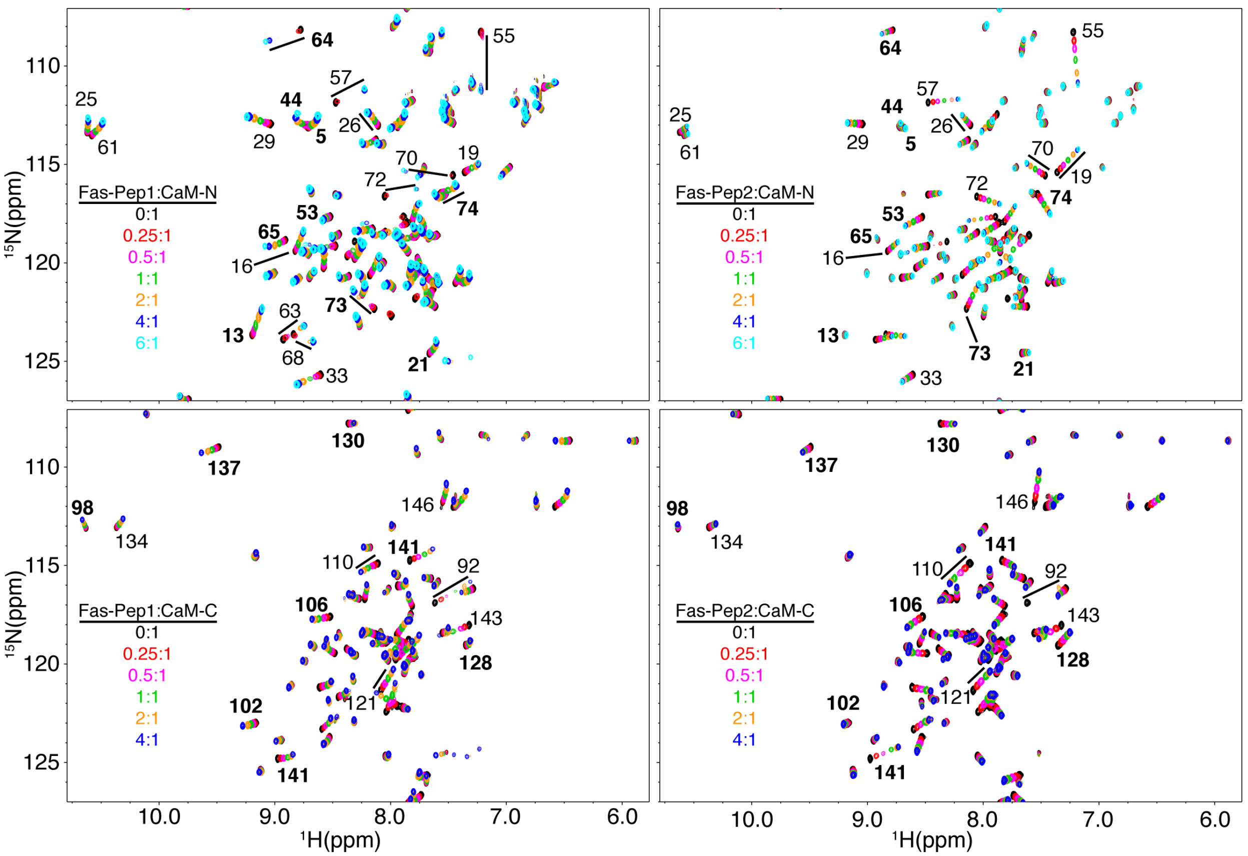

Figure S8. Overlay of 2D ^{1}H-^{15}N HSQC spectra obtained for ^{15}N-labeled Ca^{2+}/CaM-N and Ca^{2+}/CaM-C samples (150 μM) upon titration with Fas-Pep1 or Fas-Pep2. As indicated by the fast exchange on the NMR scale between free and bound states, FasDD peptides bind weaker to the isolated N and C lobes when compared to the intact Ca^{2+}/CaM protein.
