# Supplementary material for: Identification of the Calmodulin-Binding Domains of Fas Death Receptor
Source: PLoS One. 2016 Jan 6;11(1):e0146493. doi: 10.1371/journal.pone.0146493 (PMC4703387; doi:10.1371/journal.pone.0146493)
Supplement: S9 Fig — Histograms of normalized 1H-15N chemical shift changes vs. residue number calculated from the HSQC spectra for Ca2+/CaM-N and Ca2+/CaM-C complexes with Fas-Pep1 and Fas-Pep2. Notice that significant differences in chemical shift changes are observed upon binding of Ca2+/CaM-N or Ca2+/CaM-C to both peptides. For example, signals corresponding to the N-terminal residues of Ca2+/CaM-N (first 15 amino acids) exhibited substantial chemical shift changes upon binding of Fas-Pep1 (panel A). However, the 1H-15N signals corresponding to these residues were less sensitive to binding of Fas-Pep2 (panel B). Likewise, significant differences also exist in Ca2+/CaM-C residues perturbed upon binding of Fas-Pep1 vs. Fas-Pep2. Altogether, these results suggest that Fas-Pep1 and Fas-Pep2 bind to both of Ca2+/CaM-N and Ca2+/CaM-C, and that the binding mode of these peptides may be different. (PDF) [file pone.0146493.s009.pdf]

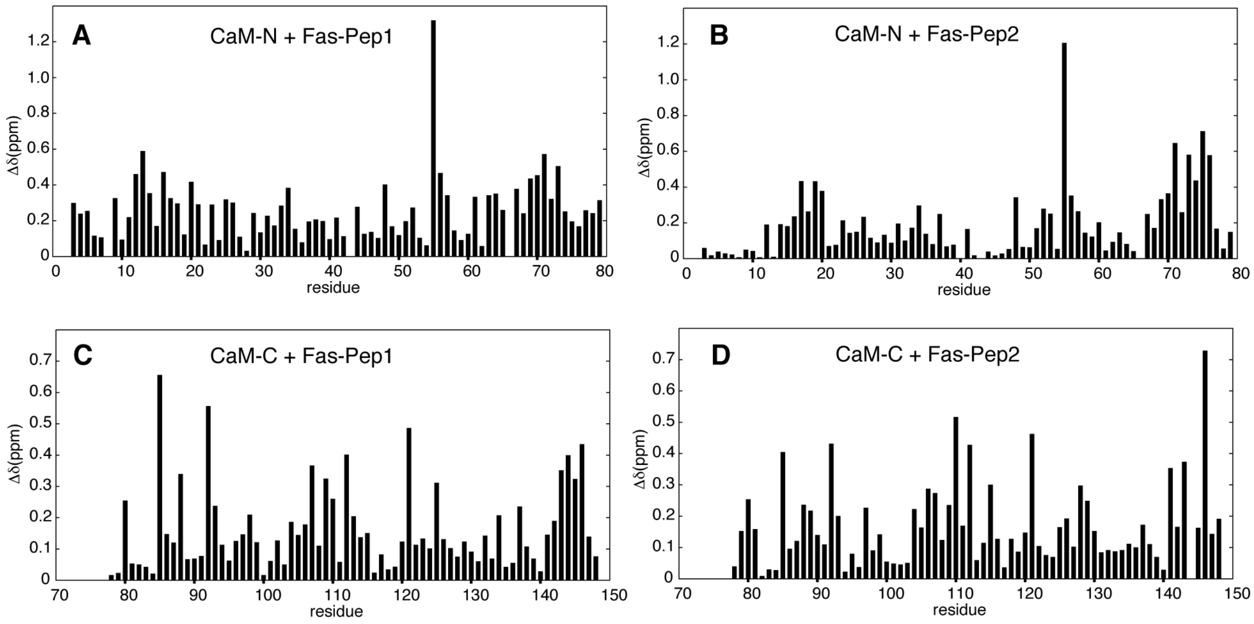

Figure S9. Histograms of normalized ^{1}H-^{15}N chemical shift changes vs. residue number calculated from the HSQC spectra for Ca^{2+}/CaM-N and Ca^{2+}/CaM-C complexes with Fas-Pep1 and Fas-Pep2. Notice that significant differences in chemical shift changes are observed upon binding of Ca^{2+}/CaM-N or Ca^{2+}/CaM-C to both peptides. For example, signals corresponding to the N-terminal residues of Ca^{2+}/CaM-N (first 15 amino acids) exhibited substantial chemical shift changes upon binding of Fas-Pep1 (panel **A**). However, the ^{1}H-^{15}N signals corresponding to these residues were less sensitive to binding of Fas-Pep2 (panel **B**). Likewise, significant differences also exist in Ca^{2+}/CaM-C residues perturbed upon binding of Fas-Pep1 vs. Fas-Pep2. Altogether, these results suggest that Fas-Pep1 and Fas-Pep2 bind to both of Ca^{2+}/CaM-N and Ca^{2+}/CaM-C, and that the binding mode of these peptides may be different.
