# Supplementary material for: Identification of the Calmodulin-Binding Domains of Fas Death Receptor
Source: PLoS One. 2016 Jan 6;11(1):e0146493. doi: 10.1371/journal.pone.0146493 (PMC4703387; doi:10.1371/journal.pone.0146493)
Supplement: S10 Fig — Binding isotherms generated by plotting the change in 1H and 15N chemical shifts (Δδ) as a function of peptide concentration. Titration data were fit by a one-site binding model. As indicated by the Kd values, FasDD peptides bind much weaker to isolated domains of Ca2+/CaM than that of the full-length protein (15–268 vs. 0.3 and 1.1 μM). Data also show that both peptides have higher affinity to Ca2+/CaM-C than Ca2+/CaM-N. (PDF) [file pone.0146493.s010.pdf]

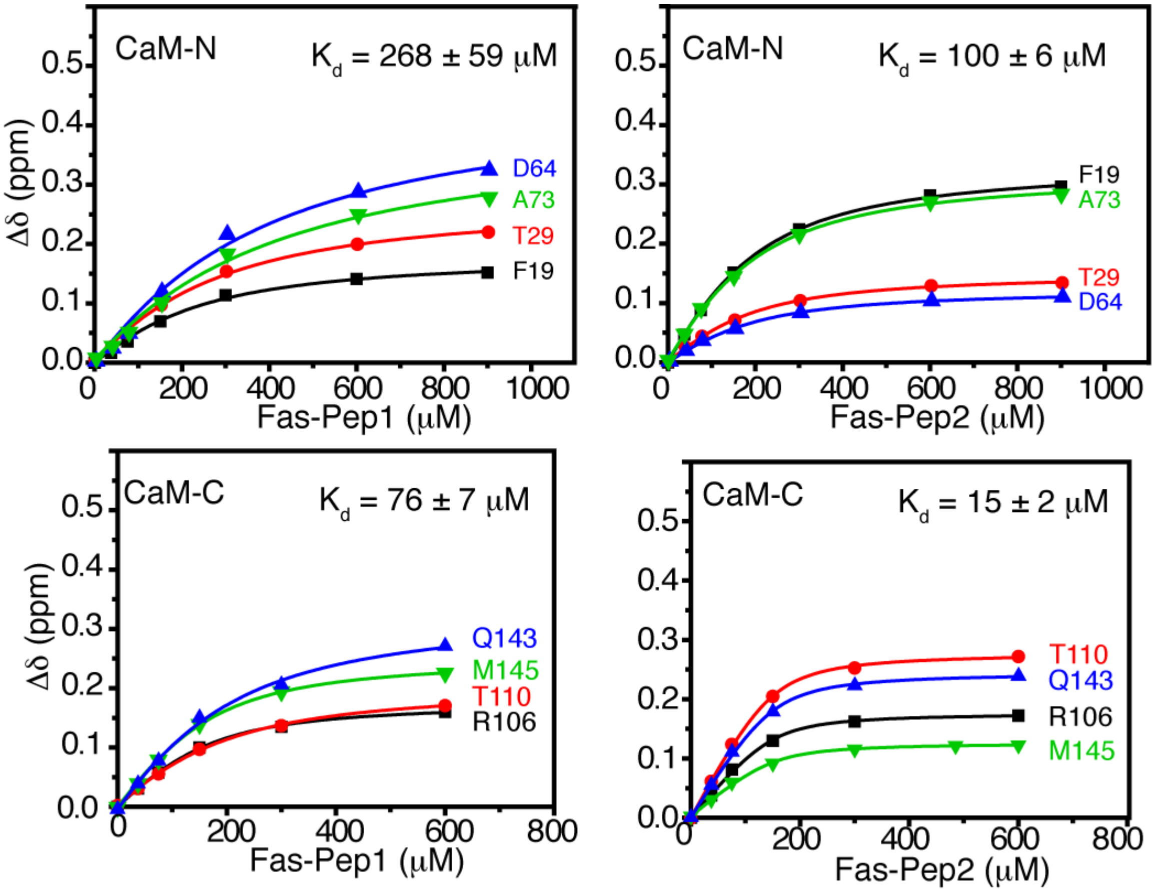

Figure S10. Binding isotherms generated by plotting the change in ^{1}H and ^{15}N chemical shifts ($\Delta\delta$) as a function of peptide concentration. Titration data were fit by a one-site binding model. As indicated by the K_d values, FasDD peptides bind much weaker to isolated domains of Ca^{2+}/CaM than that of the full-length protein (15-268 vs. 0.3 and 1.1 μM). Data also show that both peptides have higher affinity to Ca^{2+}/CaM-C than Ca^{2+}/CaM-N.
