# Supplementary material for: Identification of the Calmodulin-Binding Domains of Fas Death Receptor
Source: PLoS One. 2016 Jan 6;11(1):e0146493. doi: 10.1371/journal.pone.0146493 (PMC4703387; doi:10.1371/journal.pone.0146493)
Supplement: S1 Table — FasDD peptides obtained by subtilisin digestion and identified by mass spectrometry. (PDF) [file pone.0146493.s011.pdf]

**Table S1**. FasDD N-terminus peptides obtained by subtilisin digestion and identified by mass spectrometry.

| m/z | z | [M+H] | error (ppm) | Start | End | Peptide Sequence |
|---|---|---|---|---|---|---|
| 1190.3207 | 3 | 3568.95 | 1.91 | 205 | 236 | SAINLSDVDLSKYITTIAGVMTLSQVKGFVRKN |
| 892.9882 | 4 | 3568.93 | -2.73 | 205 | 236 | SAINLSDVDLSKYITTIAGVMTLSQVKGFVRKN |
| 714.5869 | 5 | 3568.91 | -9.9 | 205 | 236 | SAINLSDVDLSKYITTIAGVMTLSQVKGFVRKN |
| 1323.3755 | 3 | 3968.11 | -1.05 | 205 | 240 | SAINLSDVDLSKYITTIAGVMTLSQVKGFVRKNGVNE |
| 992.7859 | 4 | 3968.12 | 1.42 | 205 | 240 | SAINLSDVDLSKYITTIAGVMTLSQVKGFVRKNGVNE |
| 1347.0565 | 3 | 4039.15 | 0.422 | 205 | 241 | SAINLSDVDLSKYITTIAGVMTLSQVKGFVRKNGVNEA |
| 1010.5483 | 4 | 4039.17 | 4.49 | 205 | 241 | SAINLSDVDLSKYITTIAGVMTLSQVKGFVRKNGVNEA |
| 808.6342 | 5 | 4039.14 | -2.81 | 205 | 241 | SAINLSDVDLSKYITTIAGVMTLSQVKGFVRKNGVNEA |
| 1465.7758 | 3 | 4395.31 | -10.5 | 205 | 244 | SAINLSDVDLSKYITTIAGVMTLSQVKGFVRKNGVNEAKID |
| 1099.5876 | 4 | 4395.33 | -6.97 | 205 | 244 | SAINLSDVDLSKYITTIAGVMTLSQVKGFVRKNGVNEAKID |
| 879.8665 | 5 | 4395.3 | -12.7 | 205 | 244 | SAINLSDVDLSKYITTIAGVMTLSQVKGFVRKNGVNEAKID |
| 733.3870 | 6 | 4395.29 | -16.7 | 205 | 244 | SAINLSDVDLSKYITTIAGVMTLSQVKGFVRKNGVNEAKID |
| 1535.8230 | 2 | 3070.64 | -7.1 | 209 | 236 | SDVDLSKYITTIAGVMTLSQVKGFVRKN |
| 1024.2216 | 3 | 3070.65 | -3.35 | 209 | 236 | SDVDLSKYITTIAGVMTLSQVKGFVRKN |
| 768.4174 | 4 | 3070.65 | -4.16 | 209 | 236 | SDVDLSKYITTIAGVMTLSQVKGFVRKN |
| 1157.2780 | 3 | 3469.82 | -4.75 | 209 | 240 | SDVDLSKYITTIAGVMTLSQVKGFVRKNGVNE |
| 868.2101 | 4 | 3469.82 | -5 | 209 | 240 | SDVDLSKYITTIAGVMTLSQVKGFVRKNGVNE |
| 885.9700 | 4 | 3540.86 | -4.2 | 209 | 241 | SDVDLSKYITTIAGVMTLSQVKGFVRKNGVNEA |
